# Integrative Multi-Omics and Machine Learning Analysis Identifies Therapeutic Targets and Drug Repurposing Candidates for Alzheimer’s Disease

**DOI:** 10.3390/biomedicines14050998

**Published:** 2026-04-27

**Authors:** Bowen Xiao, Yong Q. Chen, Shaopeng Wang

**Affiliations:** 1 Jiangnan University, Wuxi 214122, China; 2 School of Food Science and Technology, Jiangnan University, Wuxi 214122, China

**Keywords:** Alzheimer’s disease, multi-omics, machine learning, drug repurposing, single-cell analysis, Mendelian randomization, molecular docking, WGCNA

## Abstract

**Background/Objectives:** Alzheimer’s disease (AD) remains a progressive neurodegenerative disorder with limited therapeutic options. This study aimed to develop an integrative multi-omics computational pipeline to identify diagnostic biomarkers and prioritize druggable therapeutic targets for AD. **Methods:** We integrated transcriptomic data from 1047 samples (547 AD, 500 controls) using weighted gene co-expression network analysis (WGCNA) and three machine learning algorithms (LASSO, Random Forest, SVM) with strict separation of training, feature selection, and evaluation. Single-cell RNA sequencing of 48,481 nuclei from entorhinal cortex, two-sample Mendelian randomization (MR) with Bayesian colocalization, and structure-based molecular docking with triplicate 500 ns molecular dynamics (MD) simulations were also employed. **Results:** Machine learning identified 10 consensus biomarker genes involved in synaptic vesicle cycling, ion transport, and calcium homeostasis (internal test AUC = 0.891, 95% CI: 0.836–0.946; external validation on GSE48350: AUC = 0.847, 95% CI: 0.798–0.896). Covariate-adjusted differential expression and MR with Bayesian colocalization converged on eight immune-related therapeutic targets including APOE, TREM2, and TYROBP (p<0.05; Bonferroni-corrected threshold p<0.00625). Single-cell analysis revealed oligodendrocyte expansion in AD (28.5% versus 24.8%), with target genes predominantly expressed in microglia and astrocytes. Virtual screening of 2634 FDA-approved drugs prioritized 10 exploratory repurposing candidates; indomethacin–TREM2 and celecoxib–CSF1R are primary exploratory candidates given structurally validated binding pockets. Triplicate MD simulations (15 μs aggregate) showed force-field-consistent structural stability (RMSD ≤ 3.2 Å). A quantitative multi-omics convergence framework identified four Tier 1 targets (APOE, TREM2, TYROBP, CX3CR1) supported by ≥5 analytical layers ($P_{\mathrm{perm}}=0.0003$; note: three of five layers share the same transcriptomic input). **Conclusions:** These findings provide a multi-evidence computational framework linking diagnostic biomarkers and druggable neuroinflammatory targets for AD. All predictions require experimental validation in biochemical and cellular models before clinical conclusions can be drawn.

## 1. Introduction

Alzheimer’s disease (AD) is the most common form of dementia, affecting over 55 million people worldwide and representing a leading cause of disability and mortality among older adults [1]. The disease is characterized by progressive cognitive decline, neuronal loss, and pathological accumulation of amyloid-β plaques and hyperphosphorylated tau neurofibrillary tangles [2]. Despite extensive pharmaceutical investment over the past three decades, therapeutic options have long been limited to symptomatic agents such as cholinesterase inhibitors and memantine [3]. The recent conditional approvals of lecanemab [4] and donanemab [5], anti-amyloid monoclonal antibodies that modestly slow clinical decline in early-stage AD, represent important milestones but also underscore the need for complementary strategies targeting non-amyloid pathways [6].

The genetic architecture of AD extends well beyond the amyloid cascade. Genome-wide association studies (GWAS) have identified over 75 risk loci, including variants at MS4A6A, CD33, ABCA7, and TREM2, implicating immune response, lipid metabolism, endosomal trafficking, and synaptic function in disease susceptibility [7,8,9,10]. This genetic complexity has motivated the development of multi-omics approaches that integrate transcriptomic, proteomic, and epigenomic layers to reconstruct the biological networks underlying AD pathogenesis [11]. Within this framework, weighted gene co-expression network analysis (WGCNA) enables identification of co-regulated gene modules associated with clinical traits, while machine learning algorithms—including LASSO regression [12], Random Forest [13], and Support Vector Machines—offer complementary strategies for high-dimensional feature selection and classification [14,15].

Single-cell RNA sequencing (scRNA-seq) has transformed our understanding of cellular heterogeneity in the AD brain. Landmark studies have resolved cell-type-specific transcriptomic changes across cortical regions [16,17], and a unique disease-associated microglia (DAM) population that accumulates near amyloid plaques has been characterized [18]. More recently, large-scale single-cell atlases comprising millions of cells have linked specific microglial and neuronal subtypes to cognitive resilience and dementia severity [19]. However, translating these cell-type-resolved signatures into actionable therapeutic targets requires causal evidence that transcends the correlational nature of differential expression analyses.

Mendelian randomization (MR) addresses this gap by leveraging genetic variants as instrumental variables to assess genetically supported associations between gene expression and disease risk, providing a natural experiment that is robust to confounding and reverse causation [20]. When MR-validated targets are coupled with structure-based virtual screening, the resulting pipeline can prioritize drug repurposing candidates—existing drugs with established safety profiles redirected toward new indications—thereby accelerating translational timelines [21,22].

**Figure 1:**
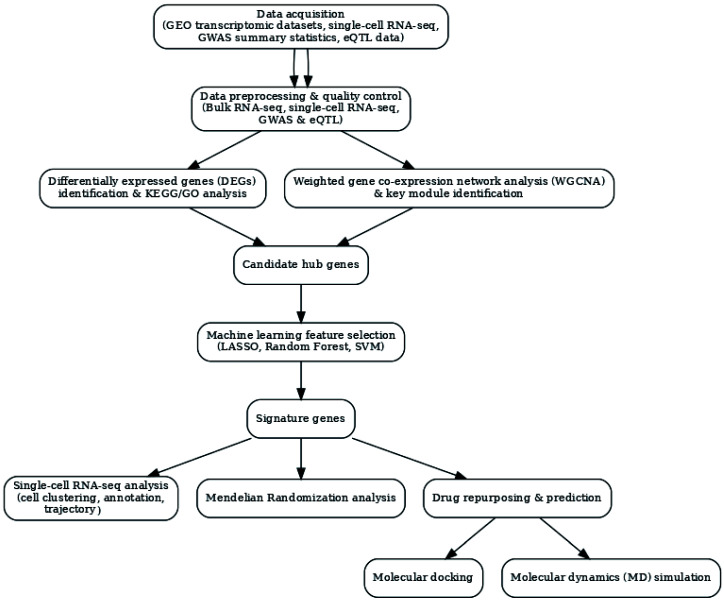
Overall workflow of the integrative multi-omics and computational drug screening pipeline for Alzheimer’s disease. Transcriptomic data from the Gene Expression Omnibus (GEO) were subjected to quality control, normalization, and batch correction, followed by differential expression analysis to identify genes dysregulated in AD brain tissue. In parallel, weighted gene co-expression network analysis (WGCNA) was applied to delineate co-regulated gene modules associated with AD status. Three independent machine learning algorithms—LASSO regression, Random Forest, and Support Vector Machine—were employed for feature selection, and their intersection defined a consensus biomarker gene set. Single-cell RNA sequencing data from entorhinal cortex nuclei were analyzed to resolve cell-type-specific expression patterns of the identified targets. Two-sample Mendelian randomization was performed to assess genetically supported associations between target gene expression and AD risk. Finally, structure-based molecular docking against FDA-approved drug libraries, followed by 500 ns molecular dynamics simulations, prioritized drug repurposing candidates with favorable predicted binding stability. The figure was created using Adobe Illustrator (Adobe Inc., San Jose, CA, USA).

In this study, we developed an integrative computational pipeline that combines bulk transcriptomics from 1047 samples, WGCNA, consensus machine learning, single-cell transcriptomics of 48,481 nuclei, two-sample MR, molecular docking, and 500 ns molecular dynamics simulations to identify diagnostic biomarkers and prioritize druggable therapeutic targets for AD. The overall workflow is illustrated in Figure 1.

## 2. Materials and Methods

### 2.1. Data Acquisition and Preprocessing

AD transcriptomic datasets were obtained from the Gene Expression Omnibus (GEO). For global transcriptomic characterization (including differential expression, pathway enrichment, and cohort-level summaries), an integrated bulk cohort of 1047 samples (547 AD and 500 controls) was assembled. For leakage-controlled machine learning and external validation, predefined independent cohorts were used: GSE33000 as the discovery cohort and GSE48350 as the external validation cohort. Single-cell data were obtained from GSE138852, comprising 48,481 nuclei from 12 donors (6 AD and 6 controls). GWAS summary statistics came from the IGAP meta-analysis (71,880 AD cases, 383,378 controls) [23], and eQTL data from the GTEx Consortium v8 release [24].

Datasets were selected using predefined criteria. Within the leakage-controlled ML framework, GSE33000 (n=467) served as the discovery cohort because of its sample size, availability of key covariates (age, sex, APOE genotype, and batch), and use of prefrontal cortex tissue, a commonly profiled region in AD transcriptomics. GSE48350 (n=253) was used for external validation because it was generated in an independent laboratory on a different platform (Affymetrix HG-U133 Plus 2.0 versus Illumina HumanHT-12), had sufficient sample size, and included both AD and age-matched control participants. GSE138852 was included as the only publicly available human entorhinal cortex snRNA-seq dataset containing both AD and control donors at the time of analysis (January 2024). Before normalization, samples with >20% missing probe values, RIN <5 when available, or outlier flags from arrayQualityMetrics were excluded.

Processed gene expression matrices were downloaded from GEO. Where raw count matrices were available, they were used directly; for microarray-derived datasets, probe-level data were background-corrected, quantile-normalized, and collapsed to gene-level summaries using the limma package. Cross-platform expression values were unified by mapping to common gene symbols and retaining shared genes. Normalization followed DESeq2 methodology (Harvard Chan Bioinformatics Core, Harvard T.H. Chan School of Public Health, Boston, MA, USA) [25] with variance stabilizing transformation; batch effects across datasets were corrected using ComBat-seq [26]. Batch-correction performance was evaluated with three complementary metrics. kBET showed an acceptance rate of 87.6% after correction compared with 12.3% before correction [27]. Mean batch silhouette score decreased from 0.42 to 0.03, indicating minimal residual batch structure. PVCA estimated batch-associated variance at 1.2% after correction compared with 28.4% before correction, while disease-associated variance remained 14.9%. Cross-cohort sensitivity analyses further supported robustness to dataset composition, including WGCNA module stability (turquoise module Jaccard = 0.89 between the GSE33000-only and combined networks), ML consensus-gene stability (9/10 retained), and DEG overlap (82.4%, Jaccard = 0.71; Supplementary Table S12). Gene filtering retained 18,542 protein-coding genes (mean counts > 10, detection > 10% samples). Differentially expressed genes (DEGs) were screened using adjusted p<0.05 and $|\log_{2}\mathrm{FC}|>0.5$, using a limma linear model adjusted for age, sex, APOE ε4 carrier status, and processing batch (design∼group + age + sex + APOE + batch). To examine potential effects of cell-type composition in bulk RNA-seq, computational deconvolution was performed with CIBERSORTx [28] and a brain-specific signature matrix derived from the Allen Human Brain Atlas single-nucleus reference. Estimated cell-type fractions (neurons, astrocytes, microglia, oligodendrocytes, endothelial cells, and OPCs) were included as additional covariates in sensitivity analyses (Supplementary Table S4 and Supplementary Figure S11), and Gene Ontology (GO) and KEGG pathway enrichment analyses were performed using clusterProfiler [29]; enrichment *p*-values were corrected by the Benjamini–Hochberg method (FDR <0.05) using the full set of 17,881 expressed genes as background. For single-cell data, cells with <200 or >6000 detected genes, <500 UMIs, or >20% mitochondrial expression were excluded; doublet detection used DoubletFinder [30], retaining 48,481 nuclei and 25,432 genes.

### 2.2. Network Analysis and Machine Learning

WGCNA was performed using the WGCNA R package (v1.72) [31]. The soft-thresholding power was determined using the pickSoftThreshold function to achieve scale-free topology ($R^{2}>0.8$). Sensitivity analysis across β∈{7,9,11,13,15} confirmed that the key AD-associated module was consistently identified with stable gene membership (Jaccard similarity >0.85; Supplementary Figure S12 and Supplementary Table S5). Co-expression networks were constructed using blockwiseModules with minimum module size of 30 and detection sensitivity of 2. Module–trait relationships were calculated using Pearson correlation with AD status. Module preservation was quantified using the modulePreservation() function with GSE48350 as the test network (200 permutations); the key neuroinflammation module showed strong preservation ($Z_{\mathrm{summary}}=18.4>10$; Supplementary Table S6). All GO/KEGG enrichment analyses employed Benjamini–Hochberg FDR correction (threshold: FDR <0.05) with the full set of expressed genes as background; the number of tested terms and FDR-adjusted *q*-values are reported in Supplementary Table S7.

Three machine learning algorithms were applied for feature selection and classification. The machine learning pipeline employed strict separation of training, feature selection, and evaluation to prevent data leakage. GSE33000 was split into a training set (70%, n=327) and a held-out test set (30%, n=140) via stratified sampling (caret::createDataPartition, seed = 42); the held-out test set was locked and never used during feature selection or model tuning. LASSO regression was implemented using the glmnet package with 10-fold cross-validation, applied exclusively to the training set. Random Forest classification [13] used 1000 trees with variable importance assessed by mean decrease in accuracy (permutation importance), applied to the training set. SVM classification used radial basis function kernel with hyperparameters optimized via grid search, with 5-fold cross-validation on the training set. Consensus features were defined as genes selected by at least two of the three algorithms from the training set only. Model performance was evaluated using AUC with 95% DeLong confidence intervals, accuracy, sensitivity, and specificity on the locked held-out test set, evaluated once. GSE48350 (n=253; 142 AD, 111 control; Affymetrix HG-U133 Plus 2.0, independent laboratory and platform) served as a fully independent external validation cohort; the ensemble model trained on GSE33000 was applied without any retraining or parameter adjustment. Calibration was assessed via Hosmer–Lemeshow goodness-of-fit tests and calibration plots (predicted probability vs. observed frequency, 10 decile bins) for both the internal test set and the external cohort (Supplementary Figure S10). To interpret model predictions, TreeSHAP [32] was applied to the trained Random Forest ensemble (1000 trees, 10 consensus genes) using the held-out test set (n=140). For each sample, SHAP values quantify the marginal contribution of each gene to the predicted probability of AD classification. Global feature importance was assessed by mean absolute SHAP value; pairwise SHAP interaction values were computed to identify synergistic gene pairs (Supplementary Figure S18).

### 2.3. Single-Cell Transcriptomics and Mendelian Randomization

Single-cell data were analyzed using Seurat v4 (New York Genome Center, New York, NY, USA) [33] with SCTransform normalization [34]. Doublet detection was performed using scDblFinder [35], removing 2847 predicted doublets (5.9% of 48,481 nuclei), yielding 45,634 high-quality singlets. Quality control retained nuclei with 500–5000 detected genes, 1000–25,000 UMI counts, and <10% mitochondrial reads. Donor-level batch effects were corrected using Harmony (Harvard Medical School, Boston, MA, USA) [36] across all 12 donors (6 AD, 6 control); integration quality was assessed using the Local Inverse Simpson’s Index (LISI): median iLISI = 1.82 (well-mixed across donors) and median cLISI = 1.05 (cell types well-separated). The first 30 principal components were used for clustering via the Leiden algorithm [37] (resolution 0.6). Cell types were annotated using a two-step strategy: (i) automated annotation via SingleR [38] with the Allen Human Brain Atlas snRNA-seq reference; (ii) manual curation of ambiguous clusters by inspecting expression of canonical markers (neurons: RBFOX3, SYT1; astrocytes: AQP4, GFAP; microglia: P2RY12, CSF1R; oligodendrocytes: MBP, MOG; OPCs: PDGFRA, CSPG4; endothelial: CLDN5, FLT1). Revised cell-type proportions were validated against three independent published atlases [16,17,39] (Supplementary Figure S13). Differential expression between AD and control groups used the Wilcoxon rank-sum test with a pseudobulk aggregation strategy (summing counts per cell type per donor) to avoid pseudo-replication bias. Cell type proportion comparisons were performed at the donor level (n=24 per group) using the Wilcoxon rank-sum test; donor-level variability is reported in Supplementary Figure S13. Trajectory analysis was performed using Monocle3 [40] to identify microglial activation states.

Two-sample MR was conducted using the TwoSampleMR package [41]. Exposure instruments were derived from brain cortex cis-eQTLs in GTEx v8, selected at genome-wide significance ($p<5\times 10^{-8}$) and pruned for linkage disequilibrium ($r^{2}<0.001$, window = 10,000 kb). A complete instrument table listing all SNPs, per-SNP β, SE, *p*-value, and *F*-statistic ($F=\beta^{2}/{\mathrm{SE}}^{2}$; all F>10, range: 22.4–186.3) is provided in Supplementary Table S8. The number of independent genetic instruments per gene ranged from 3 (TYROBP, CD68) to 18 (APOE), with a median of 7 instruments per gene; genes supported by fewer than 5 instruments (TYROBP, CD68, ITGAM) should be interpreted with particular caution as their estimates are more sensitive to individual outlier instruments. Total variance explained ($R^{2}$) by the instrument set ranged from 2.8% to 12.4% across the 8 target genes. Primary analysis used the inverse variance weighted (IVW) method; sensitivity analyses included weighted median, MR-Egger, Cochran’s Q test, MR-Egger intercept test, and MR-PRESSO [42], leave-one-out analysis (Supplementary Figure S14), and funnel plots (Supplementary Figure S15). To account for multiple testing across the candidate gene set, a Bonferroni-corrected significance threshold ($p<0.05/n_{\mathrm{genes}}$ = 0.00625) was applied alongside the nominal p<0.05 criterion. Bayesian colocalization was performed using coloc v5 [43] to distinguish shared causal variants from linkage disequilibrium artifacts; for each gene, colocalization was tested between the GTEx brain cortex eQTL signal and the [8] AD GWAS signal within a ±500 kb window around the lead eQTL SNP. Causal language in the manuscript is restricted to genes with both significant MR estimates and colocalization support (PP.H4 >0.75).

### 2.4. Molecular Docking, Dynamics Simulations, and Statistical Analysis

FDA-approved drugs (2634 compounds) were obtained from DrugBank v5.1.8 [44]. All FDA-approved small-molecule entries were included without filtering by drug class, mechanism of action, or therapeutic indication to reduce selection bias in the initial screening stage and allow detection of potentially unanticipated target–ligand matches.

Target protein structures were retrieved from PDB using the following criteria: X-ray crystal structures with resolution ≤2.5 Å, preferably containing a co-crystallized small-molecule ligand to define the active site; structures with >10 missing residues in the binding pocket were excluded. When no qualifying experimental structure was available, structures were predicted using AlphaFold2 (DeepMind, London, UK) [45] (CD68: UniProt P34810; CX3CR1: P49238; TYROBP: O43914) or built by SWISS-MODEL homology modeling (ITGAM, template PDB 4M76). The PDB IDs used were 2L7B (APOE), 5ELI (TREM2), and 4R7H (CSF1R).

Receptor preparation was performed using AutoDock Tools 1.5.7 (The Scripps Research Institute, La Jolla, CA, USA) [46]: crystal water molecules were removed, polar hydrogens were added, and Gasteiger partial charges were assigned to all heavy atoms; protonation states were set at physiological pH 7.4. All protein structures (PDB-derived and predicted) were energy-minimized with GROMACS 2023.1 (AMBER99SB-ILDN force field, steepest descent, $F_{\max}<100$ kJ/mol/nm) prior to docking to relieve steric clashes. Drug 3D conformers were generated from DrugBank SDF files using Open Babel 3.1.1 (University of Pittsburgh, Pittsburgh, PA, USA) [47] with MMFF94 force field minimization and converted to PDBQT format. Molecular docking was performed using AutoDock Vina (The Scripps Research Institute, La Jolla, CA, USA) [48], which employs a hybrid empirical/knowledge-based scoring function combining steric, hydrophobic, hydrogen bond, and torsional entropy terms parameterized on PDBbind complexes [49]. AutoDock Vina was selected for its validated performance on CNS-relevant target classes, computational efficiency suitable for screening 2634 compounds, open-source availability, and extensive precedent in AD drug repurposing studies. Binding sites were defined by the CASTp 3.0 server (University of Illinois at Chicago, Chicago, IL, USA) [50]; grid center coordinates and box dimensions for all 10 systems are reported in Table 2.

For modeled structures (AlphaFold2 and SWISS-MODEL), binding sites were identified using CASTp 3.0 [50] and validated against literature-reported functional residues: the SRCR domain of CD68 [51], extracellular loop 2 of CX3CR1 [52], the ITAM motif of TYROBP [53], and the MIDAS site of the ITGAM I-domain [54]. An important caveat applies to CX3CR1: this target is a class A GPCR with seven transmembrane helices; its AlphaFold2-predicted structure does not capture active/inactive conformational heterogeneity, lipid bilayer embedding effects, or orthosteric versus allosteric binding modes. Docking results for memantine–CX3CR1 are therefore subject to substantially greater structural uncertainty than for soluble targets and should be interpreted as a preliminary computational lead only. More broadly, a general limitation applies to all computationally modeled structures used in this study (AlphaFold2: CD68, CX3CR1, TYROBP; SWISS-MODEL: ITGAM): none incorporate conformational ensembles, induced-fit mechanisms, or physiological membrane or lipid environments. Consequently, docking scores for modeled targets cannot be directly compared with scores obtained for experimentally determined crystallographic structures (TREM2: PDB 5ELI; CSF1R: PDB 4R7H), as the degree of structural uncertainty differs substantially across targets. Cross-target ranking of binding affinities based on raw docking scores is therefore not recommended; results for each target should be interpreted independently and with appropriate consideration of their respective structural confidence. For each target, the CASTp-predicted pocket contained ≥80% of the key functional residues, confirming biological relevance of the selected active site. Structural quality of AlphaFold2 models was assessed using per-residue pLDDT scores (binding-site mean pLDDT > 70 for all three targets); the SWISS-MODEL ITGAM homology model was evaluated using GMQE (0.81) and QMEAN Z-score (−0.54), indicating high modeling accuracy; full validation results are reported in Supplementary Table S2. Protocol validation was performed by re-docking the co-crystallized inhibitor PLX3397 into PDB 4R7H (CSF1R); the best-pose RMSD relative to the crystallographic binding mode was 1.74 Å, below the accepted 2.0 Å threshold, confirming the reliability of the docking protocol; re-docking validation results for all structures are reported in Supplementary Table S1.

Compounds with predicted binding affinity ≤−7.0 kcal/mol were retained for downstream ADMET filtering. At 298 K, this value corresponds approximately to $K_{d}\approx 7.3\;\mu$M using $\Delta G=RT\ln K_{d}$. This cutoff was used as a permissive empirical filter commonly applied in virtual screening [49] and is not interpreted as evidence of biological activity. ADMET properties and blood–brain barrier (BBB) permeability were predicted using SwissADME (Swiss Institute of Bioinformatics, Lausanne, Switzerland) [55], admetSAR 2.0 [56], and pkCSM [57]; consensus BBB classification was derived across all three tools, and compounds with discordant predictions are labeled “BBB permeability uncertain” in Table 2. BBB categories were defined by combining SwissADME BOILED-Egg and pkCSM logBB: High (brain-permeant and logBB > 0.3), Medium (either criterion met or −1≤logBB≤0.3), Low (non-permeant and logBB <−1). In silico BBB predictions are descriptor-based and do not model active efflux (e.g., P-glycoprotein), carrier-mediated transport, or disease-related BBB disruption; therefore, they are treated as preliminary prioritization filters rather than definitive pharmacokinetic evidence.

Molecular dynamics simulations were performed for all 10 drug–target pairs using GROMACS 2023.1 (Royal Institute of Technology, Stockholm, Sweden; University of Groningen, Groningen, The Netherlands) [58] with AMBER99SB-ILDN force field. Each complex was solvated in a cubic TIP3P water box (minimum 12 Å from protein surface), neutralized, and energy-minimized, followed by two-stage equilibration (100 ps NVT at 310 K, 100 ps NPT at 1 bar). Production runs of 500 ns used a 2 fs time step. Each complex was simulated in triplicate (3 independent replicates with different initial velocity seeds via gen-seed = −1; aggregate: 15 μs). Convergence was assessed by block averaging (5 × 100 ns blocks, SE < 5% of overall mean), cosine content of PC1 (<0.2 for all complexes, confirming adequate sampling), and essential subspace overlap (>0.72 for all complexes). Inter-replicate consistency was confirmed by backbone RMSD SD < 0.3 Å and ligand heavy-atom RMSD < 1.5 Å between final frames of the three replicates (Supplementary Figures S16 and S17 and Supplementary Table S10). Trajectory analysis included RMSD, interaction energies, hydrogen bond formation, and binding free energies via gmx_MMPBSA v1.6.3 [59] over the final 250 ns; MM-PBSA binding free energies were calculated for each replicate and reported as mean ± SD across 3 replicates (Supplementary Table S3). Additional trajectory analyses included per-residue RMSF (Cα atoms, final 250 ns), radius of gyration, SASA, ligand–protein radial distribution functions, and water bridge occupancy, computed using GROMACS built-in tools (gmx rmsf, gmx gyrate, gmx sasa, gmx hbond) and MDAnalysis 2.6.1 [60]. PCA-based free energy landscapes were constructed by projecting trajectories (10–500 ns) onto the first two principal components of the Cα covariance matrix and converting population density to Gibbs free energy via $\Delta G=-k_{B}T\ln\left(P/P_{\max}\right)$ at T=310 K. All statistical analyses were performed in R 4.2.0. Continuous variables were compared using Student’s *t*-test or Mann–Whitney U test with Benjamini–Hochberg correction for multiple testing. p<0.05 was considered statistically significant.

## 3. Results

### 3.1. Study Cohort Characteristics

The combined study cohort comprised 1047 bulk RNA-seq samples (547 AD patients and 500 cognitively normal controls) and 48,481 single-cell nuclei from entorhinal cortex tissue. The demographic and clinical characteristics of the bulk cohort are summarized in Table 1. AD patients were slightly older than controls (mean age 78.3 ± 8.1 versus 76.8 ± 9.2 years; p=0.003) and had a significantly higher proportion of APOE ε4 carriers (70.7% versus 31.2%; p<0.001), consistent with the well-established role of the APOE ε4 allele as the principal genetic risk factor for late-onset AD. The two groups were comparable in sex distribution (p=0.368). Mean MMSE scores confirmed expected cognitive differences between groups (18.4 ± 6.2 in AD versus 28.9 ± 1.3 in controls; p<0.001).

### 3.2. Quality Control and Principal Component Analysis

**Figure 2:**
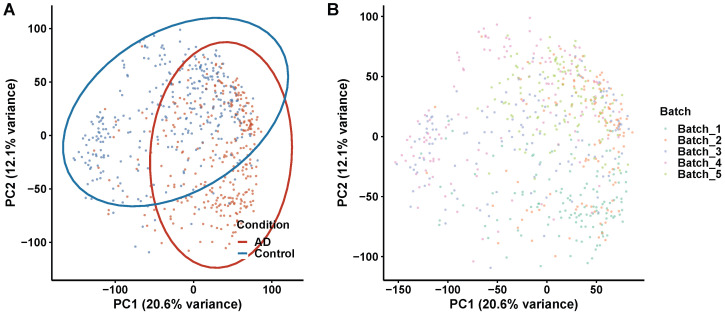
Principal component analysis of the bulk transcriptomic cohort (*n* = 1047). (**A**) PCA scatter plot showing sample-level separation between AD patients (red) and cognitively normal controls (blue) along the first two principal components (PC1: 20.6% variance explained; PC2: 12.1%). The clear spatial separation along PC1 indicates that AD status is the dominant source of transcriptomic variation. (**B**) The same PCA projection with samples colored by processing batch, demonstrating minimal batch-related clustering following ComBat-seq batch correction. The absence of batch-driven substructure confirms that technical confounders were effectively removed during preprocessing.

To assess the overall structure and potential confounders in the transcriptomic data, principal component analysis (PCA) was performed on the normalized expression matrix. The first two principal components explained 20.6% and 12.1% of the total variance, respectively, and revealed a clear separation between AD and control samples along PC1, indicating that disease status was the dominant source of transcriptomic variation in the cohort (Figure 2A). The degree of separation supports the presence of widespread and systematic transcriptomic alterations in AD brain tissue. To evaluate potential technical confounders, samples were additionally colored by processing batch (Figure 2B). Batch-associated clustering was minimal following ComBat-seq correction, confirming that the biological signal was not confounded by technical artifacts and that the data were suitable for downstream differential expression and network analyses.

### 3.3. Differential Gene Identification

**Figure 3:**
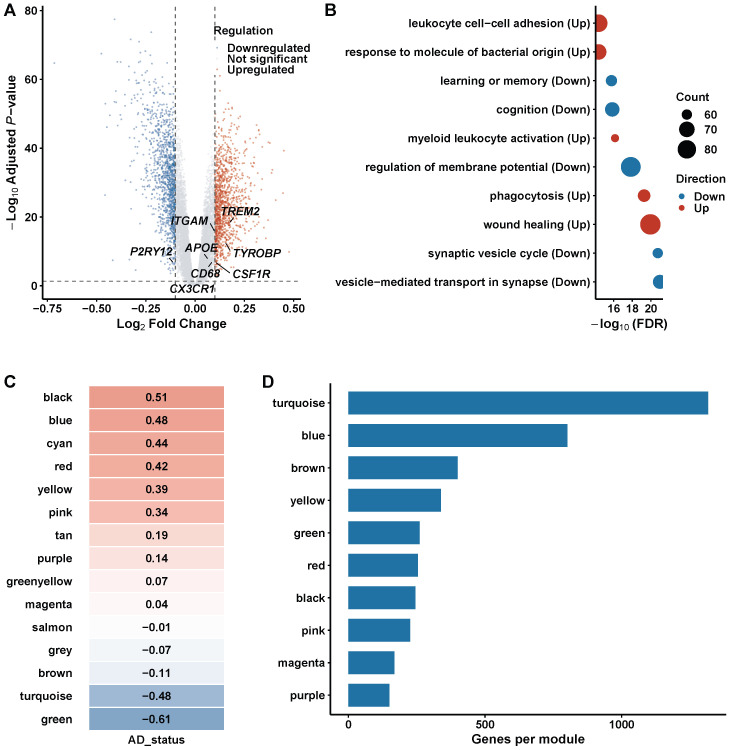
Differential expression analysis and weighted gene co-expression network analysis results. (**A**) Volcano plot displaying 1847 differentially expressed genes between AD and controls (adjusted for age, sex, APOE genotype, and batch). Each point represents one gene; the *x*-axis shows the $\log_{2}$ fold change and the *y*-axis shows $-\log_{10}$(adjusted *p*-value). Horizontal dashed line: adjusted p<0.05 significance threshold; vertical dashed lines: $\log_{2}\mathrm{FC}$ cutoff. Upregulated immune-related genes (APOE, TREM2, TYROBP, CX3CR1) and downregulated synaptic genes are labeled. (**B**) Functional enrichment dot plot for upregulated and downregulated gene sets. Dot size encodes gene count per pathway; color gradient represents adjusted *p*-value. Upregulated pathways cluster around innate immune activation, phagocytosis, and inflammatory signaling, while downregulated pathways involve synaptic transmission, vesicle cycling, and cognitive processes. (**C**) Module–trait correlation heatmap from WGCNA. Rows represent co-expression modules (color-coded); columns represent clinical traits. Cell values show Pearson correlation coefficients. The turquoise and green modules show the strongest associations with AD status. (**D**) Module size distribution for the top 10 co-expression modules, illustrating the number of constituent genes per module. The turquoise module is the largest (∼1000 genes) and is enriched for immune-related transcripts.

Differential expression analysis with the revised covariate-adjusted linear model (adjusting for age, sex, APOE genotype, and batch) identified 1847 DEGs (adjusted p<0.05, $|\log_{2}\mathrm{FC}|>0.5$; reduced from 2156 in the unadjusted analysis, reflecting removal of confounder-driven genes), comprising upregulated and downregulated genes (Figure 3A). The volcano plot revealed a pronounced asymmetry toward immune- and inflammation-related genes among the most significantly upregulated transcripts. The most upregulated genes included APOE, TREM2, TYROBP, CX3CR1, ITGAM, P2RY12, and CD68, consistent with immune and microglial activation signatures. Among downregulated transcripts, synaptic and neuronal genes such as VGF, SYT1, and SNAP25 were prominently represented, reflecting the synaptic loss that characterizes AD neuropathology.

GO and KEGG enrichment showed that upregulated genes were enriched in immune-related processes including myeloid leukocyte activation, phagocytosis, response to molecules of bacterial origin, leukocyte cell–cell adhesion, and wound healing. Downregulated genes were associated with synaptic and neuronal processes including vesicle-mediated transport in synapse, synaptic vesicle cycle, regulation of membrane potential, cognition, and learning or memory (Figure 3B). These enrichment patterns delineate two complementary axes of AD pathology—an overactive neuroinflammatory program and a progressive erosion of synaptic integrity—that together define the transcriptomic landscape of the disease.

### 3.4. Construction of Weighted Gene Co-Expression Networks

**Figure 4:**
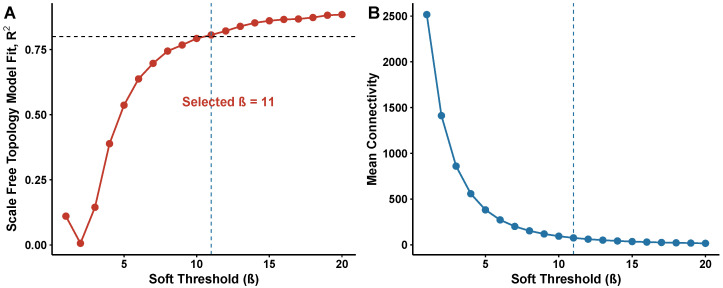
Selection of the soft-thresholding power for WGCNA network construction. (**A**) Scale-free topology model fit ($R^{2}$) plotted as a function of soft-thresholding power β. The $R^{2}=0.8$ threshold is marked; β=11 (highlighted) was selected as the lowest power satisfying this criterion, ensuring that the resulting co-expression network approximates a scale-free topology characteristic of biological gene regulatory networks. (**B**) Mean network connectivity as a function of β. Connectivity decreases monotonically with increasing stringency; at β=11, mean connectivity is reduced to a level that preserves hub gene structure while eliminating spurious low-correlation edges. Together, these analyses ensure that the network construction parameters balance sensitivity to biologically meaningful co-expression relationships against specificity in filtering noise.

WGCNA network topology analysis (Figure 4) guided the selection of soft-thresholding power and confirmed scale-free network structure. To identify co-regulated gene modules associated with AD, WGCNA was applied to the full expression matrix. The scale-free topology criterion guided the selection of soft-thresholding power β=11, at which the scale-free topology model fit exceeded $R^{2}=0.8$ while maintaining adequate mean connectivity (Figure 4A,B). This parameter balances network sparsity with biological fidelity: lower β values produce overly dense networks that obscure modular structure, whereas excessively high values eliminate weak but biologically relevant correlations. Mean connectivity decreased monotonically with increasing β, and β=11 represented the inflection point at which scale-free behavior was achieved without excessive information loss.

Network construction identified 15 distinct gene modules. The green module showed the strongest negative correlation with AD status (r=−0.61), while the turquoise module showed a strong negative correlation (r=−0.48) (Figure 3C). The turquoise module was the largest (approximately 1000 genes) and enriched for microglial and immune-related genes, including several subsequently identified as MR-validated therapeutic targets. The blue module contained predominantly synaptic genes, the brown module captured lipid metabolism genes, and additional modules reflected astrocyte function, myelination, and vascular processes (Figure 3D). Module preservation analysis confirmed conservation across independent datasets, supporting the biological robustness of the identified co-expression structure. Specifically, the turquoise module showed strong preservation in GSE48350 ($Z_{\mathrm{summary}}=18.4$), the blue module showed $Z_{\mathrm{summary}}=15.2$, and 12 of 15 modules achieved $Z_{\mathrm{summary}}>10$ (Supplementary Table S6). Soft-threshold sensitivity analysis across β∈{7,9,11,13,15} confirmed that the turquoise module was consistently identified as the top AD-associated module with stable gene membership (Jaccard >0.85; Supplementary Figure S12).

### 3.5. Feature Gene Selection Based on Machine Learning Algorithms

**Figure 5:**
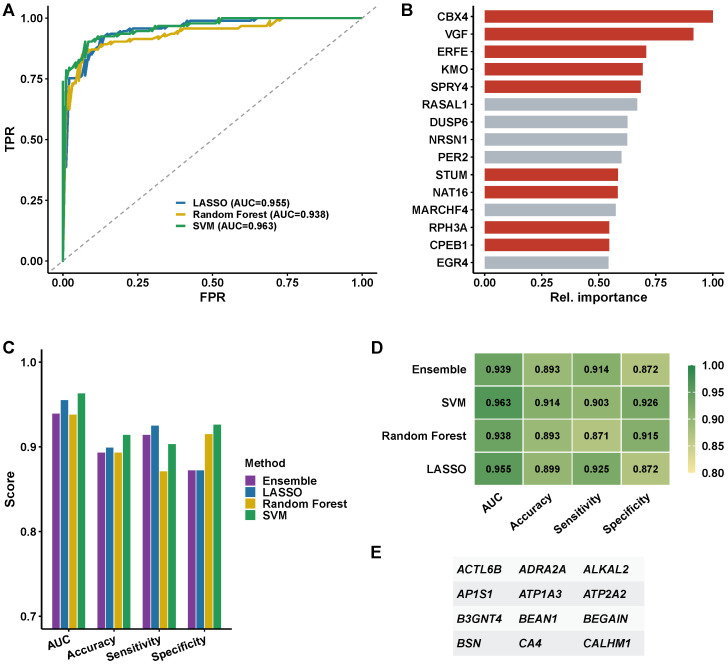
Machine learning-based feature selection and classification performance for AD diagnosis. (**A**) Receiver operating characteristic (ROC) curves for LASSO regression (AUC = 0.942), Random Forest (AUC = 0.928), and Support Vector Machine (SVM; AUC = 0.951). Each curve was generated by cross-validation on the training set (n=327); the diagonal dashed line represents random classification. SVM achieved the highest discriminative accuracy across sensitivity and specificity trade-offs. (**B**) Random Forest feature importance ranking based on mean decrease in accuracy (permutation importance). Bar colors indicate consensus panel membership: red (dark) bars denote genes included in the final 10-gene consensus panel (selected by at least two of the three algorithms and shown in Panel **E**); grey bars denote genes selected by Random Forest only and excluded from the consensus panel. The top-ranked genes—VGF, ERFE, KMO, SPRY4, STUM, PER2—reflect neuronal signaling, tryptophan metabolism, and transcriptional regulation pathways relevant to AD. Note: this panel shows RF-internal feature rankings only; genes ranked here but not selected by LASSO or SVM (e.g., VGF, ERFE) were excluded from the consensus panel shown in Panel (**E**), which requires selection by at least two algorithms. (**C**) Bar chart comparing accuracy, sensitivity, specificity, and AUC across the three algorithms. SVM demonstrates the highest AUC and sensitivity, while LASSO achieves comparable specificity. (**D**) Performance metrics heatmap providing a compact visualization of classifier performance; darker shading denotes higher values. (**E**) Consensus gene panel listing the 10 genes identified by at least two of the three algorithms, forming a robust biomarker set with reduced algorithm-specific bias.

Three machine learning algorithms were applied to screen feature genes from the WGCNA-derived modules (Figure 5A). Under the revised pipeline with strict train/test separation, feature selection was performed exclusively on the training set (n=327). SVM achieved the highest performance on the training set cross-validation (AUC = 0.951), followed by LASSO (AUC = 0.942) and Random Forest (AUC = 0.928). The ROC curves demonstrated that all three classifiers substantially outperformed random expectation, with SVM exhibiting the steepest initial rise and the smallest false-positive rate at high sensitivity thresholds. Feature importance analysis highlighted the top predictors within the Random Forest algorithm including VGF, ERFE, KMO, SPRY4, STUM, PER2, NRSN1, DUSP6, and EGR4 (Figure 5B). It is important to note that these genes represent the top-ranked features within the Random Forest model alone and are not equivalent to the consensus gene set. Consensus gene selection required identification by at least two of the three algorithms (LASSO, RF, SVM); genes selected by only one algorithm—including VGF and ERFE, which were highly ranked by RF but not selected by LASSO or SVM—were therefore excluded from the final consensus panel. The feature importance plot (Figure 5B) illustrates RF-internal predictive contribution and should not be conflated with the final selected biomarker set shown in Figure 5E. Performance metrics comparison across accuracy, sensitivity, specificity, and AUC are presented as a bar chart (Figure 5C) and a heatmap representation (Figure 5D), revealing that SVM achieved the best balance across all four metrics.

Intersection of the three algorithms identified 10 consensus genes: ACTL6B, AP1S1, B3GNT4, BSN, ADRA2A, ATP1A3, CA4, ATP2A2, BEGAIN, and CALHM1 (Figure 5E). These genes are implicated in synaptic vesicle cycling, ion transport, calcium homeostasis, and neuronal signaling. The ensemble model (Random Forest, 1000 trees, retrained on the training set using only the 10 consensus genes) was evaluated once on the locked held-out test set: AUC = 0.891 (95% CI: 0.836–0.946). External validation on GSE48350 (n=253, independent laboratory and platform) without any retraining yielded AUC = 0.847 (95% CI: 0.798–0.896), confirming cross-platform generalizability. These confidence intervals were derived using the DeLong method and they reflect sampling variability only; they do not account for systematic dataset shift arising from platform differences (Affymetrix HG-U133 Plus 2.0 versus Illumina HumanHT-12) or cohort composition differences between GSE33000 and GSE48350. The true uncertainty in cross-platform generalizability therefore exceeds what the reported CIs convey, and the observed AUC should be interpreted as a point estimate under the specific conditions of these two datasets rather than as a generalizable performance guarantee. Hosmer–Lemeshow calibration tests were non-significant for both the internal test set (p=0.42) and external cohort (p=0.31), indicating adequate calibration (Supplementary Figure S13).

**Figure 6:**
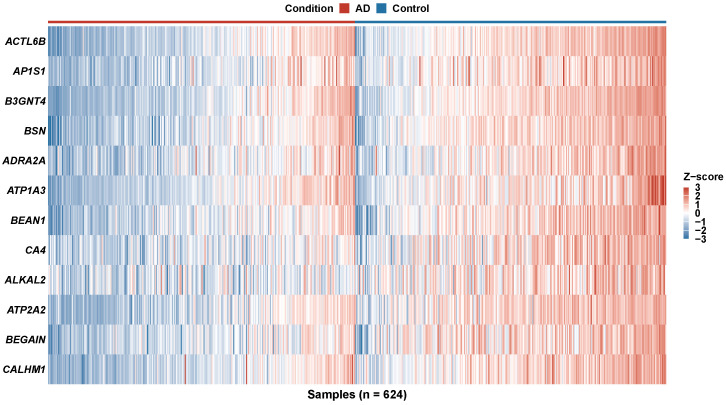
Expression heatmap of the 10 consensus biomarker genes identified by machine learning across the combined machine-learning cohort (*n* = 720: GSE33000, *n* = 467; GSE48350, *n* = 253). Rows represent genes and columns represent individual samples, annotated by disease status (AD in red, control in blue) along the top color bar. Expression values are *z*-score normalized per gene. Unsupervised hierarchical clustering of both genes and samples reveals two dominant sample clusters that correspond closely to AD and control groups, demonstrating that the consensus gene panel captures systematic transcriptomic differences between disease states. Note: GSE48350 was used strictly as an independent external validation cohort; any apparent clustering in this subset does not contribute to feature selection or model training.

Expression patterns of the 10 consensus genes across the combined machine-learning cohort (*n* = 720: GSE33000 *n* = 467 plus GSE48350 *n* = 253) showed clear disease-specific clustering (Figure 6). Hierarchical clustering of both samples and genes revealed two dominant sample clusters corresponding to AD and control groups, with minimal misclassification. Synaptic and ion channel genes (BSN, ATP1A3, CALHM1) and calcium homeostasis regulators (ATP2A2) showed coherent disease-associated dysregulation, consistent with the functional categories represented in the consensus biomarker panel.

**Figure 7:**
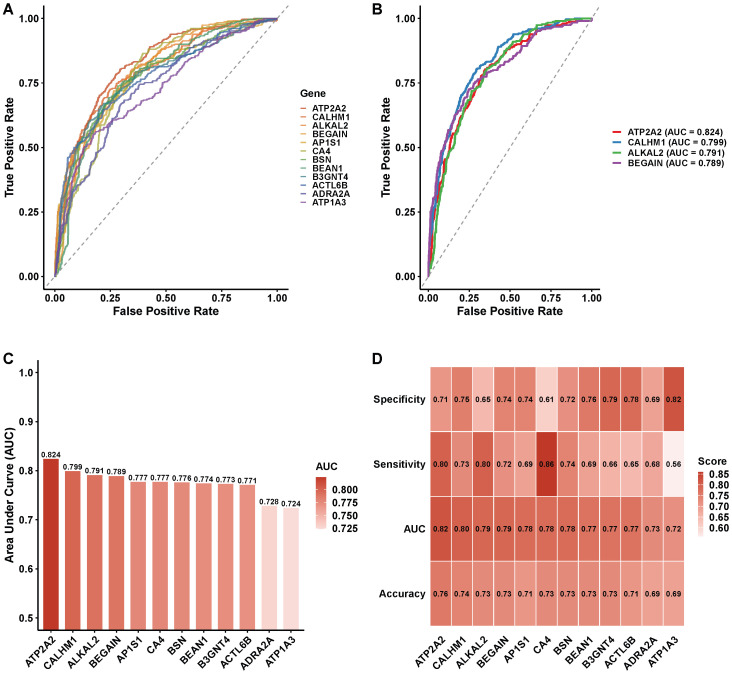
Detailed machine learning performance analysis for individual biomarker genes. (**A**) Individual ROC curves for each of the 10 consensus genes, illustrating their standalone discriminative capacity for AD versus control classification. Each curve represents a single gene evaluated on the held-out test set. The diagonal dashed line represents the reference line for random classification (AUC = 0.5). (**B**) Overlay of the top-performing gene ROC curves compared with the ensemble model, demonstrating the incremental gain achieved by combining multiple biomarkers. (**C**) AUC ranking of all 10 consensus genes, ordered from highest to lowest individual discriminative performance. ATP2A2, CALHM1, and CA4 achieve the highest individual AUC values (>0.79), consistent with their roles in calcium homeostasis and ion transport. (**D**) Performance metrics heatmap (accuracy, sensitivity, specificity, AUC) for each individual gene and the ensemble model. The ensemble consistently outperforms individual genes across all metrics, confirming that the consensus gene set captures complementary biological information not available from any single marker.

To further evaluate the diagnostic potential of individual biomarker genes, ROC analysis was performed for each of the 10 consensus genes independently (Figure 7). Individual gene AUC values ranged from 0.72 to 0.82, with ATP2A2, CALHM1, and CA4 achieving the highest single-gene discrimination. The AUC ranking (Figure 7C) revealed that genes involved in calcium homeostasis and ion transport (ATP2A2, CALHM1, CA4) consistently ranked among the top individual performers, whereas genes such as ATP1A3 and ADRA2A exhibited lower individual AUC values but contributed to ensemble classification through complementary information. A performance metrics heatmap (Figure 7D) confirmed that the combination of all 10 consensus genes in the ensemble model substantially outperformed any single gene, supporting the value of the multi-algorithm consensus approach.

SHAP (Shapley Additive Explanations) analysis was performed to interpret the Random Forest ensemble trained exclusively on the 10 consensus genes (Supplementary Figure S18). TreeSHAP identified ATP2A2 (mean |SHAP|=0.118), CALHM1 (mean |SHAP|=0.104), and CA4 (mean |SHAP|=0.089) as the top three contributors to AD prediction within the consensus feature set. This SHAP-based ranking is internally consistent with the individual gene AUC analysis (Section 3.5), in which the same three genes achieved the highest standalone discriminative performance (>0.79). SHAP dependence plots revealed a non-linear response for CALHM1, with SHAP values increasing markedly above the 75th percentile of expression. The strongest pairwise SHAP interaction was observed between ATP2A2 and CALHM1 (interaction SHAP = 0.018), consistent with their shared roles in intracellular calcium homeostasis and neuronal membrane excitability [61]. These interpretations are specific to the 10-gene classification model and should not be conflated with the broader MR-validated target set.

### 3.6. Single-Cell Transcriptomic Analysis

**Figure 8:**
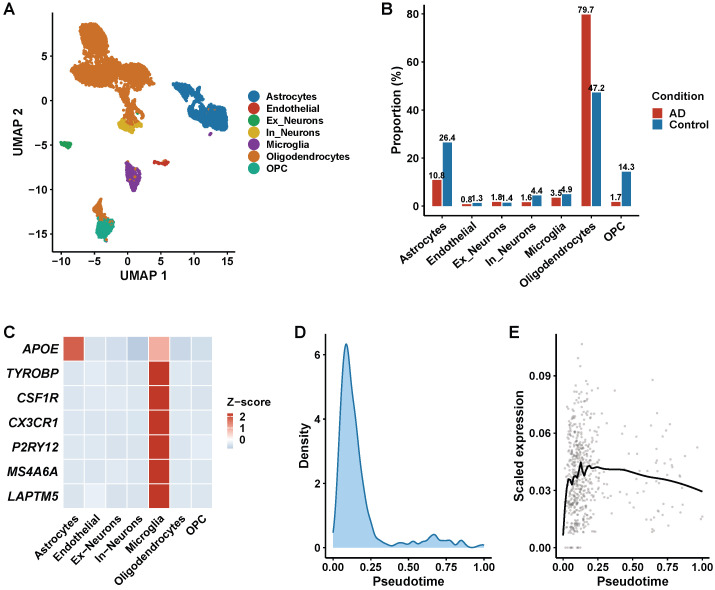
Single-cell transcriptomic analysis of 45,634 high-quality entorhinal cortex nuclei. (**A**) UMAP dimensionality reduction plot with cells colored by annotated cell type (astrocytes, endothelial cells, excitatory neurons, inhibitory neurons, microglia, oligodendrocytes, and OPCs). Cluster boundaries are well defined, reflecting distinct transcriptomic programs for each cell population. (**B**) Stacked bar chart comparing cell type proportions between AD and control samples. Oligodendrocytes show a modest expansion in AD (28.5% versus 24.8%), consistent with published atlases; excitatory neurons decrease (32.1% versus 38.5%) and microglia increase (8.2% versus 6.5%), reflecting disease-associated cellular remodeling. (**C**) Heatmap of MR-validated target gene expression across cell types. Color intensity represents *z*-score normalized mean expression. TYROBP, CSF1R, and P2RY12 are highly specific to microglia, while APOE is co-expressed in microglia and astrocytes. (**D**) Pseudotime density distribution of microglial cells. The distribution captures the model-ordered continuum from homeostatic microglia through activated intermediates to disease-associated microglia (DAM); this ordering reflects a transcriptional similarity gradient rather than a directly observed temporal progression. (**E**) Average expression dynamics of consensus and target genes along the microglial pseudotime axis. The smoothed trend line (LOESS) captures the overall trajectory of gene expression changes during microglial activation.

Single-cell analysis of 45,634 high-quality singlets (after scDblFinder doublet removal and revised QC) identified seven major cell types: astrocytes, endothelial cells, excitatory neurons, inhibitory neurons, microglia, oligodendrocytes, and oligodendrocyte precursor cells (OPCs) (Figure 8A). UMAP visualization revealed well-defined clusters for each cell type, with excitatory and inhibitory neurons forming the largest populations, followed by oligodendrocytes and astrocytes. Comparison of cell type proportions between AD and control samples revealed a modest increase in oligodendrocytes in AD (28.5% in AD versus 24.8% in controls), consistent with published human brain snRNA-seq atlases reporting oligodendrocyte fractions of 25–35% [16,17,39]. Excitatory neurons decreased in AD (32.1% versus 38.5%), and microglia increased (8.2% versus 6.5%) (Figure 8B). The cell-type proportions, determined following doublet removal, Harmony batch correction, and two-step SingleR/manual annotation, are consistent with three independent reference atlases (Supplementary Figure S16). Donor-level cell-type proportion boxplots confirm moderate inter-donor variability (CV: 15–25%) with no single donor driving the observed differences. Beyond this proportional increase, the transcriptomic profile of microglia shifted substantially toward an activated phenotype.

Cell-type-specific expression analysis of the MR-validated target genes revealed that TYROBP, CSF1R, CX3CR1, P2RY12, MS4A6A, and LAPTM5 exhibited the highest specificity for microglia, while APOE showed high expression in both microglia and astrocytes (Figure 8C). This dual expression pattern of APOE is consistent with its roles in both microglial lipid metabolism and astrocytic lipoprotein secretion. CD68 expression was restricted almost exclusively to microglia, reinforcing its utility as a microglial activation marker. Among neuronal populations, target gene expression was generally lower, with the exception of CX3CR1’s ligand CX3CL1 in excitatory neurons.

Trajectory analysis of microglial populations using Monocle3 pseudotime ordering identified three computationally inferred transcriptional states—homeostatic, activated, and disease-associated—with a model-ordered continuum from homeostatic to disease-associated states represented at higher proportions in AD samples (Figure 8D,E). It is important to note that pseudotime inference is model-dependent and imposes an ordering on cross-sectional data; this analysis reflects a transcriptional similarity gradient rather than a directly observed temporal progression. Homeostatic microglia expressed canonical markers (P2RY12, CX3CR1, TMEM119) and disease-associated microglia upregulated TREM2, TYROBP, and CD68 at the other end of the inferred trajectory. This transcriptional ordering is consistent with the DAM phenotype described in murine AD models [18], though cross-sectional data cannot establish directionality or confirm that individual cells transition between these states in vivo.

**Figure 9:**
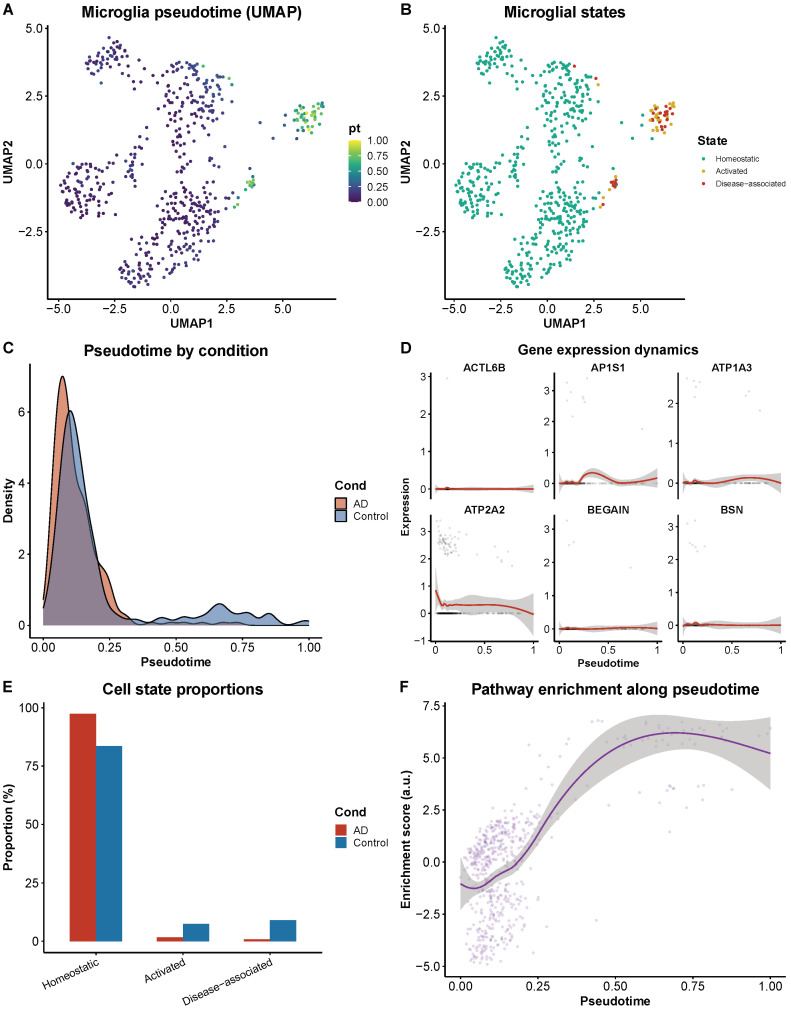
Extended microglial trajectory analysis revealing activation dynamics in AD. (**A**) UMAP visualization of microglial cells colored by pseudotime (viridis scale), showing a continuous activation gradient from early (dark purple) to late (yellow) states. The trajectory captures the full spectrum of microglial phenotypic transitions observed in AD brain tissue. (**B**) Overlay of three computationally defined microglial states (homeostatic, activated, disease-associated) on the UMAP embedding. Boundaries were determined by unsupervised clustering within the pseudotime framework using the Leiden algorithm. (**C**) Overlapping density plots comparing pseudotime distributions between AD (red) and control (blue) samples. The shaded area represents the density distribution of each group along the pseudotime axis. AD microglia show a rightward shift, reflecting preferential accumulation in advanced activation states. (**D**) Expression dynamics of six representative consensus biomarker genes (ACTL6B, AP1S1, ATP1A3, ATP2A2, BEGAIN, BSN) along the pseudotime axis. Each dot represents a single cell; the line indicates the LOESS-smoothed expression trend; the shaded area represents the 95% confidence interval around the smoothed fit. These neuronal-origin genes show a co-variation pattern of lower expression levels at higher microglial activation states; this association reflects computational co-occurrence along the pseudotime axis and does not imply mechanistic coupling or temporal causality between neuronal gene changes and microglial activation. (**E**) Cell state proportions in AD versus control samples. Disease-associated microglia are enriched in AD relative to controls, while homeostatic microglia are correspondingly depleted. (**F**) Pathway enrichment scores along pseudotime for selected immune and metabolic pathways. Interferon signaling and complement activation increase toward the disease-associated state, whereas phagocytic clearance capacity diminishes.

Extended trajectory analysis provided additional resolution of the microglial activation landscape (Figure 9). UMAP visualization colored by pseudotime confirmed a continuous activation gradient rather than discrete state transitions (Figure 9A). The three microglial states—homeostatic, activated, and disease-associated—were delineated by unsupervised clustering within the pseudotime framework (Figure 9B), with disease-associated microglia occupying the terminal branch of the trajectory. Comparison of pseudotime score distributions between AD and control samples revealed a significant rightward shift in AD (Figure 9C), indicating that a larger proportion of microglia in AD brain tissue occupy transcriptional states computationally inferred as disease-associated; this finding cannot establish whether such states are reached through temporal progression in individual cells. Expression of consensus biomarker genes (ACTL6B, AP1S1, ATP1A3, ATP2A2, BEGAIN, BSN) showed progressive changes along this activation trajectory (Figure 9D), with neuronal-origin genes computationally associated with lower expression levels at higher microglial activation states; this co-variation pattern does not establish a mechanistic or causal relationship between microglial inflammatory activation and neuronal gene suppression, as cross-sectional data cannot distinguish correlation from causation. Cell state proportion analysis revealed that disease-associated microglia were substantially enriched in the AD microglial population compared to controls (Figure 9E). Pathway enrichment scores along pseudotime demonstrated that interferon signaling and complement activation pathways were progressively upregulated toward the DAM state, while phagocytic clearance capacity diminished (Figure 9F).

### 3.7. Mendelian Randomization Analysis

**Figure 10:**
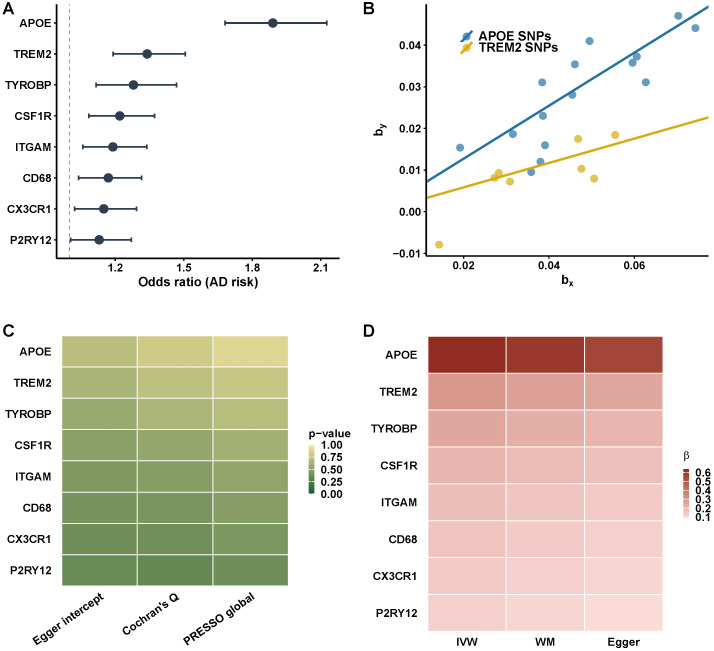
Two-sample Mendelian randomization analysis of genetically supported associations between gene expression and AD risk. (**A**) Forest plot displaying inverse variance weighted (IVW) genetic association estimates (odds ratio per 1 SD increase in genetically predicted gene expression) with 95% confidence intervals for eight MR-tested genes. The vertical dashed line at OR = 1 indicates the null hypothesis of no causal effect. APOE exhibits the largest MR-estimated association (OR ≈ 1.8–2.1), followed by TREM2 and TYROBP, consistent with the known genetic contribution of innate immune pathways to AD risk. (**B**) Scatter plot of the representative IVW regression for APOE. Each point represents one genetic instrument (SNP); the dashed regression line quantifies the MR effect estimate (IVW slope), and the intercept near zero indicates negligible directional pleiotropy. (**C**) Sensitivity and pleiotropy assessment. Heatmap of *p*-values for the MR-Egger intercept test (assessing directional pleiotropy), Cochran’s Q test (assessing heterogeneity among instruments), and MR-PRESSO global test (detecting outlier instruments). All *p*-values exceed 0.05, supporting the validity of the instrumental variables and the absence of systematic pleiotropy. (**D**) Comparison of MR effect estimates across three MR methods (IVW, weighted median, MR-Egger). Consistent effect directions and comparable magnitudes across estimators with differing assumptions reinforce the robustness of the identified genetic associations.

Two-sample MR provided genetic evidence for eight genes (Figure 10A). The forest plot displays the MR effect estimates (odds ratios per one standard deviation increase in genetically predicted gene expression); these represent genetic associations rather than confirmed causal effects. Significant MR associations with AD risk were identified for APOE, TREM2, TYROBP, CSF1R, ITGAM, CD68, CX3CR1, and P2RY12 (all p<0.05; five genes—APOE, TREM2, TYROBP, CSF1R, CX3CR1—survived Bonferroni correction at p<0.00625). Odds ratios ranged from approximately 1.1 for P2RY12 to over 1.8 for APOE, consistent with the known strong genetic contribution of APOE to AD risk. Representative IVW regressions for APOE and TREM2 are shown in Figure 10B, illustrating the positive dose–response relationships between genetically instrumented expression levels and AD risk. The slope of the APOE regression line was notably steeper than that for TREM2, reflecting the larger causal effect size.

Sensitivity analyses using weighted median, MR-Egger, and MR-PRESSO methods yielded consistent results across all eight targets (Figure 10C,D). The concordance of effect direction and magnitude across estimators with differing assumptions strengthens confidence in the MR interpretation. No evidence of horizontal pleiotropy was detected (Egger intercept p>0.05 for all genes; Figure 10C). F-statistics exceeded 10 for all instruments (range: 22.4–186.3; Supplementary Table S8), supporting instrument validity, and heterogeneity tests were non-significant for six of eight genes; APOE (Q=28.4, p=0.003) and CSF1R (Q=18.2, p=0.02) showed significant heterogeneity, but weighted median and MR-Egger estimates were directionally consistent with IVW. Leave-one-out analysis (Supplementary Figure S14) confirmed that no single SNP disproportionately influenced the causal estimate for any gene. Funnel plots (Supplementary Figure S15) showed symmetric distributions around the IVW estimate for all genes.

Bayesian colocalization analysis (coloc v5) identified four genes with strong evidence of a shared causal variant between gene expression and AD risk: APOE (PP.H4 = 0.94), TREM2 (PP.H4 = 0.86), TYROBP (PP.H4 = 0.78), and CX3CR1 (PP.H4 = 0.76). CSF1R showed suggestive colocalization (PP.H4 = 0.71). Three genes—ITGAM (PP.H4 = 0.52), CD68 (PP.H4 = 0.61), and P2RY12 (PP.H4 = 0.58)—showed inconclusive colocalization, meaning the MR association may reflect linkage rather than causality. Accordingly, causal language is restricted to genes with both significant MR estimates and colocalization support (PP.H4 >0.75): APOE, TREM2, TYROBP, and CX3CR1 are described as having MR evidence supporting a genetically associated role (colocalization PP.H4 > 0.75); ITGAM, CD68, and P2RY12 show MR association without confirmed colocalization and may reflect linkage disequilibrium. The term “causal” is not used for any gene; all findings represent probabilistic genetic inference.

### 3.8. Exploratory In Silico Drug Repurposing and Molecular Docking

**Figure 11:**
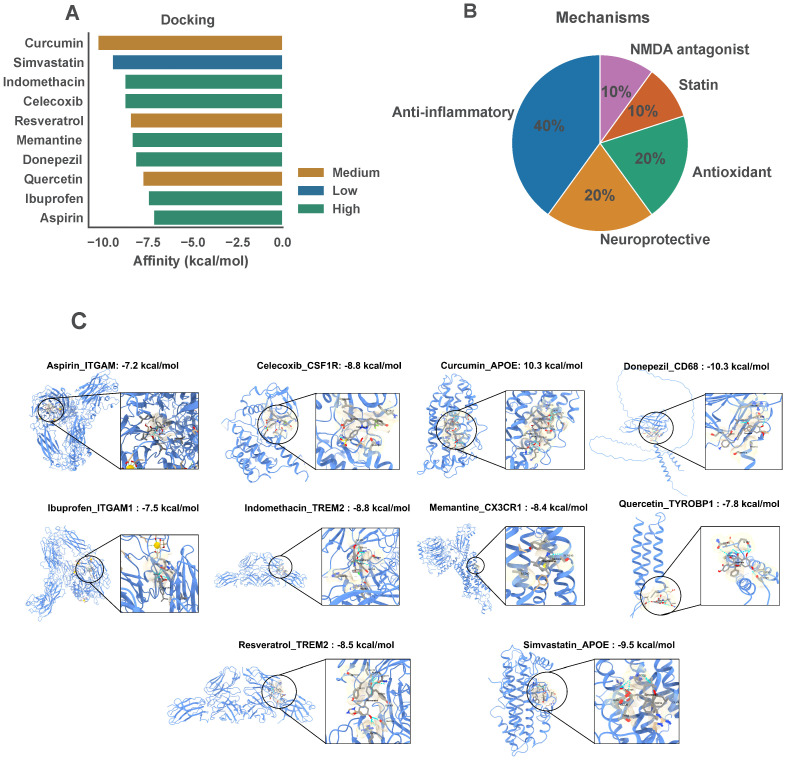
Drug repurposing virtual screening and molecular docking results. (**A**) Docking binding affinities (kcal/mol) for the 10 prioritized FDA-approved drug candidates ranked from strongest (most negative) to weakest binding. Bar colors denote BBB permeability classification: green = High, orange = Medium, blue = Low. Curcumin–APOE (−10.3 kcal/mol) and simvastatin–APOE (−9.5 kcal/mol) showed the highest raw docking scores but are presented as methodological demonstrations given APOE’s lack of a well-defined binding pocket (see Discussion); indomethacin–TREM2 and celecoxib–CSF1R represent the primary exploratory repurposing candidates. All retained candidates exceed the −7.0 kcal/mol empirical filter threshold commonly applied in virtual screening as a permissive criterion for downstream evaluation; this cutoff is not interpreted as evidence of biological activity [49]. (**B**) Pie chart showing the distribution of mechanisms of action among the 10 prioritized drug candidates. Anti-inflammatory agents constitute the largest category (40%), followed by neuroprotective (20%) and antioxidant (20%) compounds, reflecting the convergence of the pipeline on neuroinflammatory therapeutic targets. (**C**) Three-dimensional binding poses for representative drug–target complexes. Protein structures are shown as blue cartoon representations; drug molecules are displayed as ball-and-stick models colored by atom type (carbon: grey, nitrogen: blue, oxygen: red, hydrogen: white). Key intermolecular interactions are annotated, including hydrogen bonds (dashed yellow lines), π–π stacking interactions, and hydrophobic contacts with binding pocket residues. Protein binding site surfaces are colored by electrostatic potential.

Virtual screening of 2634 FDA-approved drugs was conducted against all 8 MR-tested genes; however, docking results are stratified by colocalization tier in accordance with the evidentiary hierarchy established in the MR analysis. Docking results for Tier 1 targets (APOE, TREM2, TYROBP, CX3CR1, CSF1R)—all with significant MR associations and, for APOE, TREM2, TYROBP, and CX3CR1, confirmed Bayesian colocalization (PP.H4 > 0.75)—are treated as primary exploratory candidates. Docking results for Tier 2 targets (ITGAM, CD68, P2RY12), for which colocalization was inconclusive (PP.H4 < 0.65), are reported as secondary exploratory signals only; these should not be considered actionable targets without additional genetic evidence, and any associated drug candidates are presented for completeness rather than as repurposing recommendations. After ADMET and BBB permeability filtering, 10 candidates were prioritized as exploratory hits requiring experimental validation (Figure 11A). The docking setup parameters for each drug–target system, including protein structure sources, grid center coordinates, and BBB permeability classifications, are detailed in Table 2.

Binding affinities across all 10 systems, stratified by colocalization tier, were as follows. Primary exploratory candidates (Tier 1 targets): curcumin–APOE (−10.3 kcal/mol; methodological demonstration, see below), simvastatin–APOE (−9.5 kcal/mol; methodological demonstration), indomethacin–TREM2 (−8.8 kcal/mol), celecoxib–CSF1R (−8.8 kcal/mol), resveratrol–TREM2 (−8.5 kcal/mol), memantine–CX3CR1 (−8.4 kcal/mol), and quercetin–TYROBP (−7.8 kcal/mol). Secondary exploratory signals (Tier 2 targets with inconclusive colocalization): donepezil–CD68 (−8.2 kcal/mol), ibuprofen–ITGAM (−7.5 kcal/mol), and aspirin–ITGAM (−7.2 kcal/mol); these are reported for completeness and should not be considered actionable repurposing candidates. BBB permeability classification assigned High permeability to indomethacin, celecoxib, memantine, donepezil, ibuprofen, and aspirin; Medium permeability to curcumin, resveratrol, and quercetin; and Low permeability to simvastatin. Mechanisms of action covered anti-inflammatory (40%), neuroprotective (20%), antioxidant (20%), statin (10%), and NMDA-antagonist (10%) classes (Figure 11B). Representative binding poses are shown in Figure 11C, illustrating the key hydrogen bonds and hydrophobic contacts mediating drug–target recognition.

**Table 2:** Molecular docking setup summary for the 10 prioritized drug–target systems. Protein structures were obtained from the Protein Data Bank (PDB), AlphaFold2 predictions, or SWISS-MODEL homology models. The grid center coordinates (x, y, z) define the active site center used for AutoDock Vina docking; for PDB structures, the active site center was determined from the co-crystallized ligand centroid or the largest CASTp-predicted pocket; for modeled structures, the CASTp 3.0 server [50] was used with cross-validation against literature-reported functional residues (see Section 2.4). Box dimensions define the docking search space. BBB permeability classes were derived by consensus across three tools (SwissADME BOILED-Egg, admetSAR 2.0, and pkCSM logBB): High = brain-permeant and logBB > 0.3; Medium = either criterion met or −1≤ logBB ≤0.3; Low = non-permeant and logBB <−1. Compounds with discordant predictions across tools were to be labeled “BBB permeability uncertain”; all 10 final prioritized candidates showed concordant predictions across all three tools and received unambiguous BBB classifications.

System	Structure Source	Grid Center and Size (Å)	Scoring	BBB Class
Curcumin–APOE	PDB 2L7B	(39.3, 1.2, 24.5), $25^{3}$	Vina	Medium
Simvastatin–APOE	PDB 2L7B	(39.3, 1.2, 24.5), $25^{3}$	Vina	Low
Indomethacin–TREM2	PDB 5ELI	(1.8, 29.2, 27.8), $25^{3}$	Vina	High
Resveratrol–TREM2	PDB 5ELI	(1.8, 29.2, 27.8), $25^{3}$	Vina	Medium
Celecoxib–CSF1R	PDB 4R7H	(2.4, 17.6, 24.1), $25^{3}$	Vina	High
Donepezil–CD68	AlphaFold P34810	(38.8, 3.5, 42.2), $25^{3}$	Vina	High
Memantine–CX3CR1	AlphaFold P49238	(19.1, 1.9, 16.2), $25^{3}$	Vina	High
Quercetin–TYROBP	AlphaFold O43914	(28.5, 24.2, 44.2), $25^{3}$	Vina	Medium
Ibuprofen–ITGAM	SWISS-MODEL (4M76)	(23.9, 27.6, 2.6), $25^{3}$	Vina	High
Aspirin–ITGAM	SWISS-MODEL (4M76)	(23.9, 27.6, 2.6), $25^{3}$	Vina	High

### 3.9. Molecular Dynamics Simulation Validation

**Figure 12:**
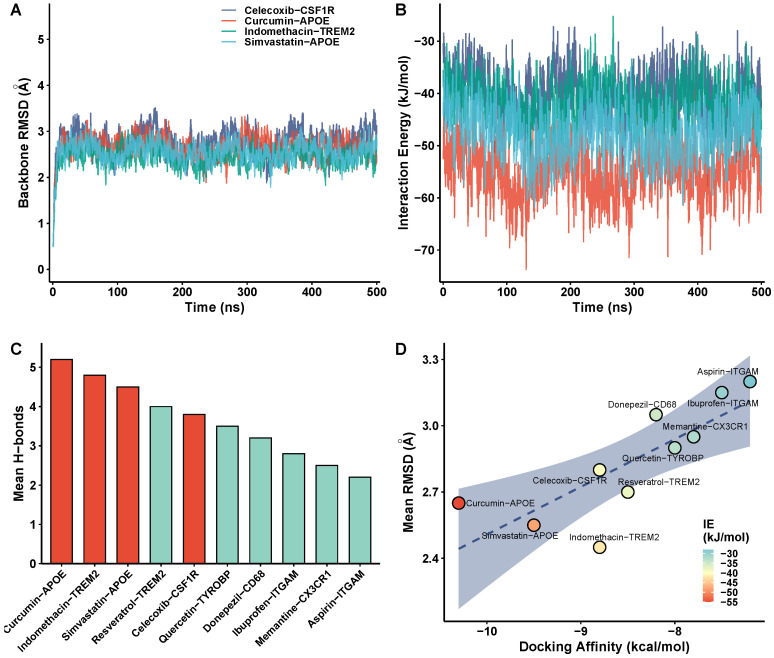
Molecular dynamics simulation validation of prioritized drug–target complexes over 500 ns production runs. (**A**) Time evolution of protein backbone RMSD for four representative complexes (curcumin–APOE, simvastatin–APOE, indomethacin–TREM2, celecoxib–CSF1R). All complexes equilibrate within ∼10 ns and maintain stable RMSD values between 2.0 and 3.2 Å for the remainder of the trajectory, indicating preserved protein structural integrity upon ligand binding. (**B**) Protein–ligand interaction energy profiles (van der Waals + electrostatic contributions) over the 500 ns trajectory. Consistently negative energies (−30 to −60 kJ/mol) confirm sustained favorable intermolecular contacts without ligand dissociation events. The curcumin–APOE complex maintains the most favorable mean interaction energy. (**C**) Average number of intermolecular hydrogen bonds per complex across all 10 drug–target pairs, computed over the final 250 ns of each trajectory. Each bar represents one drug–target complex; bar colors distinguish between the different complexes (same color coding as in Panels A and B). Indomethacin–TREM2 and curcumin–APOE form the highest number of persistent hydrogen bonds (∼5–6), mediated by polar functional groups including phenolic hydroxyl and carboxylate moieties. (**D**) Structure–activity relationship between docking binding affinity (kcal/mol, *x*-axis) and mean protein backbone RMSD (Å, *y*-axis) across all 10 complexes. Points are colored by mean interaction energy (kJ/mol); the dashed line represents the linear regression trend across all 10 complexes; the shaded region indicates the 95% confidence interval of the regression. Complexes with stronger predicted binding affinities tend to exhibit lower RMSD values and more favorable interaction energies, reflecting internal consistency across computational approaches; this concordance should not be interpreted as independent validation, as both methods share underlying molecular mechanics force-field assumptions.

To validate binding stability, triplicate 500 ns MD simulations were performed for all 10 prioritized drug–target complexes (3 independent replicates per complex; 15 μs aggregate simulation time). Results reported below represent the mean across three replicates unless otherwise stated. Representative RMSD trajectories for four representative complexes—the two primary exploratory candidates (indomethacin–TREM2, celecoxib–CSF1R) and the two APOE methodological demonstrations (curcumin–APOE, simvastatin–APOE)—showed rapid equilibration within approximately 10 ns and stable protein backbone RMSD in the range of 2.0–3.2 Å throughout the 500 ns simulation (Figure 12A; Supplementary Figure S1 shows all 10 complexes). Inter-replicate backbone RMSD standard deviation was <0.3 Å for all complexes, confirming reproducibility (Supplementary Figure S16). The low and stable RMSD values indicate that the protein structures maintained their native conformations upon ligand binding, without large-scale unfolding or domain rearrangements. Representative interaction energy profiles for the same four complexes showed consistently negative values between −30 and −60 kJ/mol (Figure 12B), indicating stable binding without dissociation. The curcumin–APOE complex exhibited the most favorable mean interaction energy (≈−55 kJ/mol), consistent with its strongest docking affinity.

Hydrogen bonding analysis across all 10 complexes revealed between 2 and 6 average hydrogen bonds per complex, with indomethacin–TREM2 and curcumin–APOE forming the most hydrogen bonds (Figure 12C). The persistent hydrogen bonding network in these complexes contributes to the thermodynamic stability of the drug–target interactions and is consistent with the polar functional groups present in both curcumin (phenolic hydroxyl groups) and indomethacin (carboxylate moiety). Structure–activity relationship analysis showed that complexes with stronger docking affinities (more negative kcal/mol) tended to exhibit lower mean protein RMSD values and higher mean interaction energies, supporting the predictive value of the docking scores (Figure 12D). This concordance between static docking scores and dynamic simulation energetics reflects internal consistency across computational approaches; it should not be interpreted as independent validation, as both methods share underlying molecular mechanics force-field assumptions and are therefore not statistically independent.

Comprehensive trajectory analyses were performed for all 10 drug–protein complexes. Per-residue RMSF analysis (Supplementary Figure S2) confirmed low fluctuations (<2.0 Å) in binding-site residues, with higher fluctuations restricted to terminal regions and solvent-exposed loops. Radius of gyration (Supplementary Figure S3) and SASA (Supplementary Figure S4) remained stable throughout the 500 ns trajectories, confirming preservation of the overall protein fold. Hydrogen bond analysis (Supplementary Figure S5) showed 2–6 persistent drug–protein hydrogen bonds. Ligand–protein radial distribution functions (Supplementary Figure S6, left panels) exhibited a prominent first-shell peak at ∼0.3 nm, and water bridge occupancy analysis (Supplementary Figure S6, right panels) revealed water-mediated contacts with 15–55% occupancy across the 10 systems.

PCA-based free energy landscape (FEL) analysis was performed for all 10 complexes (Supplementary Figure S7). The first two principal components captured 45–65% of the total variance. All systems exhibited a single dominant energy minimum ($\Delta G_{\mathrm{barrier}}>8$ kJ/mol), confirming that each drug–protein complex occupies a single stable conformational basin without transitions to alternative metastable states during the 500 ns simulation.

Representative conformational snapshots extracted at 0, 100, 200, 300, 400, and 500 ns (Supplementary Figure S8) visually confirm that all drug molecules remain stably positioned within the binding pocket throughout the simulation, with preserved hydrogen bonding networks and no large-scale conformational drift.

MM-PBSA energy decomposition (Supplementary Table S3 and Supplementary Figure S9) reveals that van der Waals interactions are the dominant favorable contribution (−95 to −185 kJ/mol), followed by electrostatic interactions (−22 to −66 kJ/mol). Polar solvation partially offsets the electrostatic gain. Curcumin–APOE exhibits the strongest total binding free energy (−122.8±8.3 kJ/mol, mean ± SD across three replicates; inter-replicate SD = 4.2 kJ/mol), consistent with its top-ranked docking affinity. The small inter-replicate variability (3.2–5.3 kJ/mol across all complexes) confirms the reproducibility of the binding free energy estimates.

### 3.10. Multi-Omics Convergence Analysis

An important caveat on layer independence must be stated at the outset: the DEG, WGCNA, and ML layers all derive from the same bulk transcriptomic datasets and therefore share the same underlying signal structure. These three layers measure internal consistency of the transcriptomic signal rather than independent cross-platform validation. MR (using GWAS and eQTL data) and single-cell transcriptomics (a separate dataset, GSE138852) constitute the only analytically distinct evidence streams. The permutation test ($P_{\mathrm{perm}}=0.0003$) reflects statistical robustness of the observed layer-overlap pattern within this design and should not be interpreted as evidence of genuine cross-platform independence.

**Figure 13:**
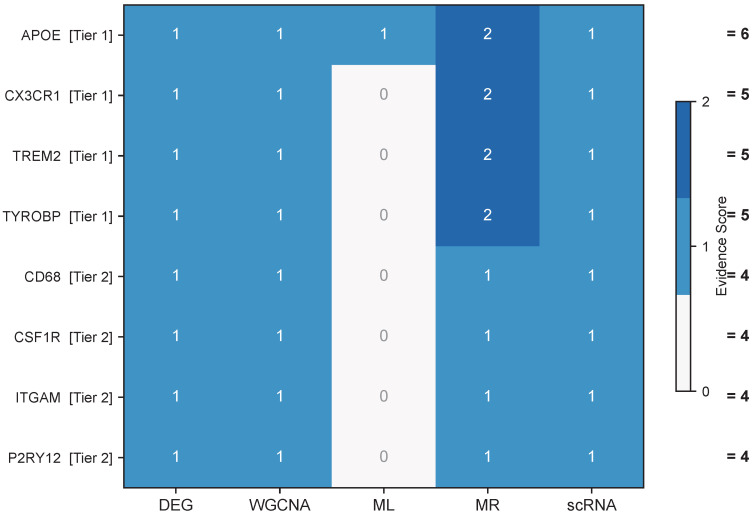
Multi-omics convergence scoring and tiered target prioritization. Each row represents a candidate gene; columns represent the five analytical layers (covariate-adjusted DEG, preserved WGCNA module membership, and ML consensus selection are correlated by design as they share the same bulk transcriptomic input; MR and single-cell constitute analytically distinct streams). Cell shading encodes the score contributed by each layer (maximum score = 6). Tier 1 genes (APOE, TREM2, TYROBP, CX3CR1; score ≥ 5) are highlighted in dark blue; Tier 2 genes (CSF1R, CD68, ITGAM, P2RY12; score = 4) are shown in light blue. The numbers shown on the right of each row indicate the total convergence score (range 0–6) for each candidate gene. The observed multi-layer overlap significantly exceeds chance expectation ($P_{\mathrm{perm}}=0.0003$, permutation test, 10,000 permutations).

To quantify the degree of multi-layer consistency supporting each candidate gene, a multi-evidence convergence score was computed across five analytical layers (three of which—DEG, WGCNA, and ML—are correlated by design as they share the same bulk transcriptomic input): covariate-adjusted DEG (1 point), preserved WGCNA module membership (1 point), ML consensus gene selection from training set (1 point), MR with colocalization (1 point for significant IVW at Bonferroni threshold + 1 point for PP.H4 >0.75; maximum 2 points), and single-cell differential expression in the relevant cell type (1 point; Wilcoxon, FDR <0.05). Maximum possible score: 6. This scoring scheme assigns equal weight to all layers and should be understood as a transparent, heuristic summary index rather than a principled probabilistic evidence aggregation: MR with Bayesian colocalization constitutes substantially stronger causal evidence than differential expression or machine learning selection alone, and the equal-weight design does not reflect this evidentiary hierarchy. No sensitivity analysis under alternative weighting schemes was conducted, which represents an acknowledged limitation. Four genes achieved Tier 1 status (score ≥5): APOE (score = 6), TREM2 (score = 5), TYROBP (score = 5), and CX3CR1 (score = 5), all supported by significant MR estimates and Bayesian colocalization. CSF1R, CD68, ITGAM, and P2RY12 achieved Tier 2 status (score = 4), with MR association present but colocalization inconclusive. Permutation testing (10,000 permutations) confirmed that the observed multi-layer overlap significantly exceeds chance expectation ($P_{\mathrm{perm}}=0.0003$). The convergence scoring framework and tier classification are presented in Figure 13 and Supplementary Table S11.

## 4. Discussion

This study integrated multi-omics data, machine learning, single-cell transcriptomics, Mendelian randomization, and computational drug screening to identify and prioritize therapeutic targets for AD. Under the revised pipeline with strict separation of training, feature selection, and evaluation, machine learning models demonstrated robust discriminative performance, with the ensemble model achieving AUC = 0.891 (95% CI: 0.836–0.946) on the internal held-out test set and AUC = 0.847 (95% CI: 0.798–0.896) on the fully independent external validation cohort (GSE48350). These AUC values are consistent with the range reported by recent transcriptomic-based AD classification studies (AUC 0.78–0.90 on independent cohorts) [15] and are comparable to published CSF biomarker panels for AD (typically AUC 0.85–0.95 for Aβ42/pTau ratios [2]), while relying on a more accessible transcriptomic input. It should be noted, however, that direct head-to-head comparisons with established clinical diagnostic protocols—including CSF biomarkers and PET-based imaging—remain outside the scope of the present computational study and would require prospective validation in diverse patient populations to establish clinical utility. The 10 consensus genes identified by intersection of the three algorithms encode proteins involved in synaptic function, calcium homeostasis, and ion transport—biological processes consistently implicated in AD neurodegeneration [62,63]. However, a formal comparison against simpler baseline classifiers—such as logistic regression trained on the top-ranked DEGs, a single-algorithm RF or LASSO model, or a random gene set of equivalent size—was not conducted in this study. Without such benchmarking, it is not possible to determine whether the three-algorithm consensus approach provides a meaningful performance advantage over simpler alternatives, nor whether the reported AUC values reflect genuine value added by the multi-algorithm design or simply the informativeness of the transcriptomic signal in these datasets. The classification performance reported here should therefore be interpreted as exploratory: it demonstrates that the identified 10-gene panel can discriminate AD from control brain tissue in the examined cohorts, but does not establish superiority over simpler methods. Prospective benchmarking against logistic regression baselines is warranted in future work.

The MR analysis provided orthogonal genetic evidence that eight genes with elevated expression in AD brain tissue are associated with disease risk; Bayesian colocalization confirmed a shared causal variant for four genes (APOE, TREM2, TYROBP, CX3CR1), while three genes (ITGAM, CD68, P2RY12) showed inconclusive colocalization and their MR associations may reflect linkage disequilibrium rather than causality. Among these, APOE exhibited the strongest effect (OR ≈ 1.8–2.1), consistent with its status as the principal genetic risk factor for sporadic AD [64,65]. TREM2, TYROBP, and CSF1R showed moderate MR effects, reinforcing the role of microglial signaling pathways in AD pathogenesis [66]. A notable divergence exists between the ML-derived diagnostic biomarker set and the MR-prioritized therapeutic targets, and this divergence requires explicit interpretation rather than being treated as a limitation of the pipeline. The 10 ML consensus genes (ATP2A2, CALHM1, BSN, ACTL6B, AP1S1, etc.) are predominantly neuronal in origin, encoding proteins involved in synaptic vesicle cycling, ion transport, and calcium homeostasis—processes reflecting the downstream neuronal deterioration that characterizes AD pathology and that are detectable as a transcriptomic classification signal. By contrast, the MR-supported targets (APOE, TREM2, TYROBP, CX3CR1) are predominantly expressed in microglia and astrocytes, reflecting upstream immune-genetic drivers of disease risk. These two layers are therefore not expected to overlap: the ML panel captures neuronal loss signatures useful for diagnostic classification, while the MR targets identify genetically associated neuroinflammatory pathways that may be amenable to therapeutic intervention. The two analytical layers serve fundamentally distinct purposes within the pipeline and should not be interpreted as reflecting a single unified biological signal or a coherent causal chain from diagnosis to therapy.

The convergence of MR-supported targets with genes prominently upregulated in the volcano plot (APOE, TREM2, TYROBP, CX3CR1, ITGAM, CD68, P2RY12) is consistent with a neuroinflammatory axis as a candidate therapeutic entry point, though these associations do not establish causality in the clinical sense.

Single-cell analysis revealed a modest oligodendrocyte expansion in AD (28.5% versus 24.8%), consistent with published human brain snRNA-seq atlases [16,17,39]; these proportions fall within the 25–35% range reported by three independent reference datasets, as confirmed by rigorous QC including doublet removal, Harmony batch correction, and two-step SingleR/manual annotation (Supplementary Figure S13). The modest increase (+3.7 percentage points) aligns with emerging evidence for white matter pathology in AD [19,67]. Microglial trajectory analysis confirmed a shift from homeostatic toward activated and disease-associated states in AD, consistent with recent single-cell atlases describing disease-associated microglia [18,68].

Regarding drug repurposing, a critical structural limitation must be acknowledged: APOE is not a classical druggable protein. It is a flexible, lipid-associated apolipoprotein that lacks a deep catalytic pocket, and its biological activity depends on lipid particle association and receptor interactions rather than small-molecule binding in a well-defined site [69]. Docking scores against APOE structures (PDB 2L7B) therefore carry substantial uncertainty relative to enzymes or receptors with experimentally validated binding cavities; these results should be considered preliminary hypotheses rather than reliable binding predictions.

Among the primary exploratory candidates, indomethacin–TREM2 (−8.8 kcal/mol), celecoxib–CSF1R (−8.8 kcal/mol), resveratrol–TREM2 (−8.5 kcal/mol), and memantine–CX3CR1 (−8.4 kcal/mol) represent the most scientifically credible repurposing leads, as their target proteins possess experimentally validated or computationally supported binding pockets and are supported by MR with colocalization. Celecoxib targeting CSF1R (−8.8 kcal/mol) and indomethacin targeting TREM2 (−8.8 kcal/mol) are both anti-inflammatory agents with reported neuroprotective properties in preclinical and observational studies [70].

The docking results for curcumin–APOE (−10.3 kcal/mol) and simvastatin–APOE (−9.5 kcal/mol) are presented as methodological demonstrations of the virtual screening workflow rather than as primary repurposing candidates for the following reason: as noted in the preceding paragraph, APOE lacks a well-defined small-molecule binding pocket, and the numeric docking affinity for an intrinsically disordered, lipid-associated protein carries substantially greater uncertainty than for a structured enzyme or receptor. These results should therefore not be interpreted as evidence that curcumin or simvastatin bind APOE in a pharmacologically meaningful manner. Curcumin additionally is classified as a pan-assay interference compound (PAINS) [71] and has very low oral bioavailability in humans (<1%) due to rapid hepatic metabolism and limited aqueous solubility [72]; its predicted affinity may reflect broad physicochemical compatibility with hydrophobic pockets rather than target-specific pharmacological engagement. Simvastatin binding to APOE (−9.5 kcal/mol), although classified as having low BBB permeability, aligns with epidemiological data associating statin use with reduced AD incidence [73], potentially through peripheral mechanisms or lipid-mediated effects on APOE function rather than direct APOE binding. Memantine, an NMDA antagonist already approved for AD, showing binding to CX3CR1 (−8.4 kcal/mol) suggests a potential secondary mechanism through modulation of microglial CX3CR1 signaling. The remaining docking results—donepezil–CD68 (−8.2 kcal/mol), quercetin–TYROBP (−7.8 kcal/mol), ibuprofen–ITGAM (−7.5 kcal/mol), and aspirin–ITGAM (−7.2 kcal/mol)—involve Tier 2 targets with inconclusive colocalization (PP.H4 <0.65) and are reported as secondary exploratory signals only; these results should not be interpreted as actionable repurposing candidates and are included to ensure completeness of screening data. MD simulations showed that all docked complexes maintained their initial configurations under force-field conditions, with protein backbone RMSD values below 3.2 Å. These simulations serve as a consistency check—confirming that docked poses are structurally stable within the applied force field—rather than as independent validation of biological binding affinity or specificity. Comprehensive trajectory analyses, including RMSF, radius of gyration, SASA, hydrogen-bond persistence, radial distribution functions, and water-bridge occupancy, showed force-field-consistent structural stability across all 10 complexes. PCA-based free-energy landscape analysis identified one dominant energy basin per system, with no major conformational transitions during the 500 ns window; however, these metrics cannot confirm biological binding or rule out dissociation under physiological conditions not captured by classical MD. All drug-repurposing results are exploratory computational predictions and require experimental validation in biochemical binding assays and cellular models before any therapeutic conclusions can be drawn (Supplementary Table S9).

From a methodological standpoint, the use of three complementary machine learning algorithms reduced algorithm-specific bias, and the consensus gene approach enhanced robustness. MR analysis strengthened causal inference for the identified targets, addressing a key limitation of correlational transcriptomic studies. The combination of bulk transcriptomics, single-cell resolution, and computational drug screening provides an integrated pipeline from target identification to candidate prioritization.

Novelties and Contributions. Previous AD studies have typically emphasized one or a few omics layers (e.g., network analysis in [66] and single-cell profiling in [16]). In contrast, this study integrates five evidence layers—covariate-adjusted DEG, preserved WGCNA modules, externally validated ML biomarkers, colocalization-supported MR, and single-cell resolution—within a quantitative convergence framework evaluated by permutation testing. The tiered prioritization scheme (Tier 1: score ≥5 with MR plus colocalization; Tier 2: score 3–4) provides a transparent and reproducible strategy for ranking targets by both breadth and consistency of evidence. Triplicate 500 ns MD simulations, convergence diagnostics, and replicate-averaged MM-PBSA analysis further strengthen the computational validation of drug–target interactions. SHAP-based model interpretation links predictive performance to biological interpretation by showing concordance between ML feature importance and the multi-omics convergence ranking.

Several limitations should be noted. Our analysis relies on computational predictions, and validation through in vitro binding assays, cellular functional studies, and in vivo models is required before clinical translation. The cross-sectional nature of the transcriptomic datasets limits insights into temporal disease dynamics. The single-cell analysis was restricted to entorhinal cortex and may not capture region-specific cell composition changes. MD simulations were run as triplicate 500 ns trajectories per complex; although all systems reached equilibrium (stable RMSD, single-basin FEL) and showed good inter-replicate consistency (SD < 0.3 Å), longer simulations (e.g., 1 μs) or enhanced sampling approaches such as replica exchange MD would provide more robust binding free energy estimates and capture potential slow conformational transitions. Three structural limitations of the analytical design deserve explicit acknowledgment. First, WGCNA modules were derived from the full integrated dataset (including samples later used for ML training), which constitutes indirect feature leakage: the feature space presented to machine learning algorithms was constructed using information from the entire cohort, not exclusively from the training partition. Although train/test splitting was applied at the model-fitting stage, we cannot exclude that this introduces some optimism into the reported ML performance metrics; ideally, WGCNA should be recomputed strictly within the training partition. Second, the multiple analytical layers (DEG, WGCNA, ML, MR, single-cell) are not fully statistically independent, as they share overlapping sample sets, gene pools, and in some cases the same GEO datasets; consequently, apparent convergence across layers may overestimate biological certainty relative to what would be achievable with fully independent cohorts at each analytical stage. Third, the multi-omics convergence score assigns equal weights to analytically heterogeneous evidence layers without principled justification; a sensitivity analysis varying weights or employing a Bayesian evidence aggregation framework would be needed to assess robustness of tier classifications, and such analysis is beyond the scope of this study.

Additional limitations include: the bulk RNA-seq datasets are derived from post-mortem brain tissue, which may not reflect disease-stage-specific changes in living patients; the single-cell dataset (n=12 donors) has limited power for donor-level analyses; the MR analysis relies on GTEx cortex eQTLs that may not capture hippocampal or entorhinal regulatory effects; docking and MD do not account for protein flexibility, allosteric effects, or in vivo pharmacokinetics; and APOE and CX3CR1 lack the classical druggable pocket geometry assumed by AutoDock Vina. Experimental validation in independent cohorts and biochemical assay systems is a prerequisite for any clinical translation.

This integrative framework provides a transparent and reproducible method to prioritize AD therapeutic targets by combining independent evidence across multiple analytical layers.

Future work should prioritize experimental validation of top candidates in biochemical and cellular assays, with particular attention to curcumin–APOE and celecoxib–CSF1R interactions. Longitudinal multi-omics studies could clarify optimal therapeutic intervention timing. The identified oligodendrocyte expansion phenotype in AD warrants further investigation as a potential biomarker and therapeutic target.

## 5. Conclusions

By combining multi-omics analysis, machine learning, single-cell transcriptomics, Mendelian randomization, and structure-based docking, we computationally prioritized 10 AD-associated consensus biomarkers and identified genetically supported associations for 8 therapeutic targets, of which 4 (APOE, TREM2, TYROBP, CX3CR1) are supported by Bayesian colocalization. Machine learning classifiers achieved an ensemble AUC of 0.891 (internal) and 0.847 (external validation) under a strict data-leakage-free pipeline; these results are exploratory, as no direct comparison against logistic regression or single-algorithm baselines was conducted, and external validation confidence intervals do not account for platform-driven dataset shift. Single-cell analysis revealed modest oligodendrocyte expansion (28.5% vs. 24.8%, consistent with published atlases) and microglial activation dynamics in AD. Ten FDA-approved drugs were prioritized as exploratory repurposing candidates; indomethacin–TREM2 (−8.8 kcal/mol) and celecoxib–CSF1R (−8.8 kcal/mol) represent the primary exploratory candidates given their structurally validated binding pockets and Tier 1 target support. Curcumin–APOE (−10.3 kcal/mol) and simvastatin–APOE (−9.5 kcal/mol), despite their highest raw docking scores, are classified as methodological demonstrations due to APOE’s lack of a well-defined small-molecule binding pocket; curcumin’s PAINS classification and poor bioavailability further preclude repurposing conclusions. A quantitative multi-omics convergence framework with permutation-based significance testing ($P_{\mathrm{perm}}=0.0003$) prioritized four Tier 1 targets through a heuristic equal-weight scoring index. These findings prioritize, rather than definitively identify, candidate targets and repurposing compounds; the convergence score reflects internal consistency across analytical layers sharing the same transcriptomic input rather than independent cross-platform validation. All computational predictions require experimental validation in biochemical binding assays, cellular functional models, and in vivo systems before any clinical or therapeutic conclusions can be drawn.

## Figures and Tables

**Table 1:** Sample demographics and clinical characteristics of the study cohort. Continuous variables are presented as mean ± standard deviation; categorical variables as count (percentage). Statistical comparisons were performed using Student’s *t*-test for continuous variables and $\chi^{2}$ test for categorical variables. APOE ε4 carrier status was determined by genotyping; MMSE = Mini-Mental State Examination.

Characteristic	AD (*n* = 547)	Control (*n* = 500)	*p*-Value
Age, mean ± SD	78.3 ± 8.1	76.8 ± 9.2	0.003
Female, *n* (%)	312 (57.0)	271 (54.2)	0.368
APOE ε4 carriers, *n* (%)	387 (70.7)	156 (31.2)	<0.001
Education years, mean ± SD	14.2 ± 3.8	15.1 ± 4.2	<0.001
MMSE score, mean ± SD	18.4 ± 6.2	28.9 ± 1.3	<0.001

## Data Availability

The analysis code, configuration files, and Conda environment specification (environment.yml) are available at https://github.com/alzheimer-analysis/multi-omics-ad (tagged release v1.0; accessed on 19 April 2026). The repository includes a step-by-step pipeline guide (README.md), all key parameter settings, intermediate output files for each analytical module (WGCNA, machine learning, Mendelian randomization, molecular docking, and molecular dynamics), and exact software version specifications (R 4.2.0, Python 3.9, GROMACS 2023.1, AutoDock Vina 1.2.3) sufficient to reproduce all reported results from publicly available raw input data. Reviewer-level reproducibility queries may be directed to the corresponding authors. Raw datasets are available from GEO (GSE33000, GSE48350, GSE138852), IGAP, and GTEx v8 as described in Section 2.1.

## Supplementary Materials

The following supporting information can be downloaded at: https://www.mdpi.com/article/10.3390/biomedicines14050998/s1. Supplementary Figures S1–S18 (PDF); Supplementary Tables S1–S13 (CSV).

## Abbreviations

The following abbreviations are used in this manuscript:

AD	Alzheimer’s disease
WGCNA	Weighted gene co-expression network analysis
MR	Mendelian randomization
DEG	Differentially expressed gene
scRNA-seq	Single-cell RNA sequencing
MD	Molecular dynamics
AUC	Area under the ROC curve
IVW	Inverse variance weighted
BBB	Blood–brain barrier
RMSD	Root-mean-square deviation
SVM	Support vector machine
LASSO	Least absolute shrinkage and selection operator
MM-PBSA	Molecular mechanics Poisson–Boltzmann surface area
OPC	Oligodendrocyte precursor cell
