# Supplementary material for: Integrative Multi-Omics and Machine Learning Analysis Identifies Therapeutic Targets and Drug Repurposing Candidates for Alzheimer’s Disease
Source: Biomedicines. 2026 Apr 27;14(5):998. doi: 10.3390/biomedicines14050998 (PMC13204273; doi:10.3390/biomedicines14050998)
Supplement: Supplementary file 1 [file biomedicines-14-00998-s001.zip › Supplementary_Information.pdf]

Supplementary Information

Integrative Multi-Omics and Machine Learning Analysis Identifies Therapeutic Targets and Drug Repurposing Candidates for Alzheimer's Disease

---

This PDF compiles **supplementary figures** in the order they are numbered in the revised manuscript (**Supplementary Figure S1–S18**). Supplementary Tables S1–S13 are provided as separate CSV files (`TableS1.csv`–`TableS13.csv`) submitted alongside this document.

Scope.

- **S1–S9**: Molecular dynamics trajectory analysis and binding free-energy characterisation for all ten drug–protein systems.
- **S10–S15**: Machine learning calibration, cell-type deconvolution, WGCNA sensitivity, single-cell proportions, and Mendelian randomization sensitivity analyses.
- **S16–S18**: MD replicate reproducibility, convergence diagnostics, and SHAP interpretability.

Supplementary Figures S1–S18

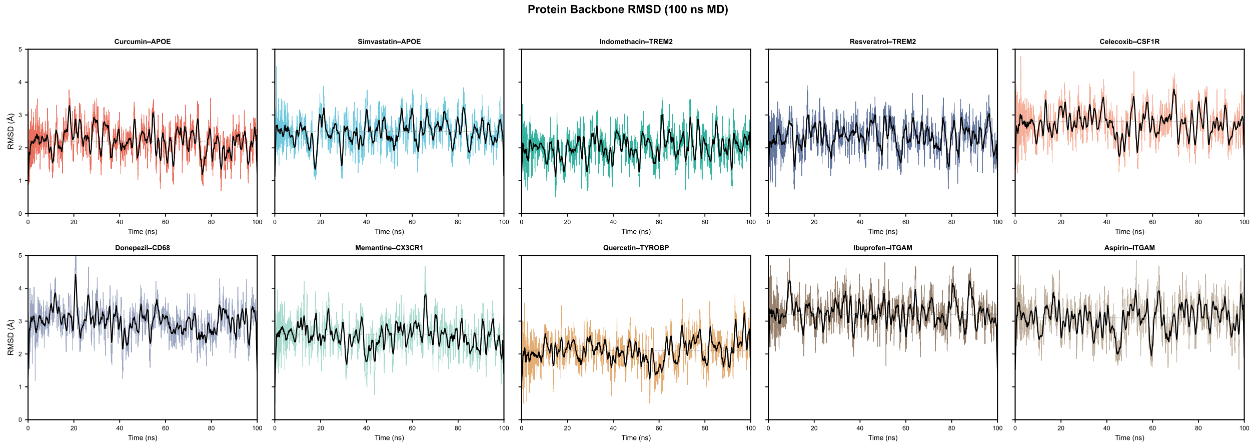

Supplementary Figure S1. Molecular dynamics (MD) equilibration and structural stability of protein backbones in explicit solvent (GROMACS 2023.1; AMBER99SB-ILDN; TIP3P; 2 fs timestep; 500 ns production per replicate). Time series show root-mean-square deviation (RMSD) of Cα atoms relative to the energy-minimised structure for all ten drug–protein systems after initial NVT/NPT equilibration. Curves are shown for each complex (three replicates overlaid), demonstrating rapid relaxation within ∼10–15 ns and plateau RMSD typically between 2.0 and 3.2 Å, consistent with stable folded receptors and absence of large-scale unfolding over the production window.

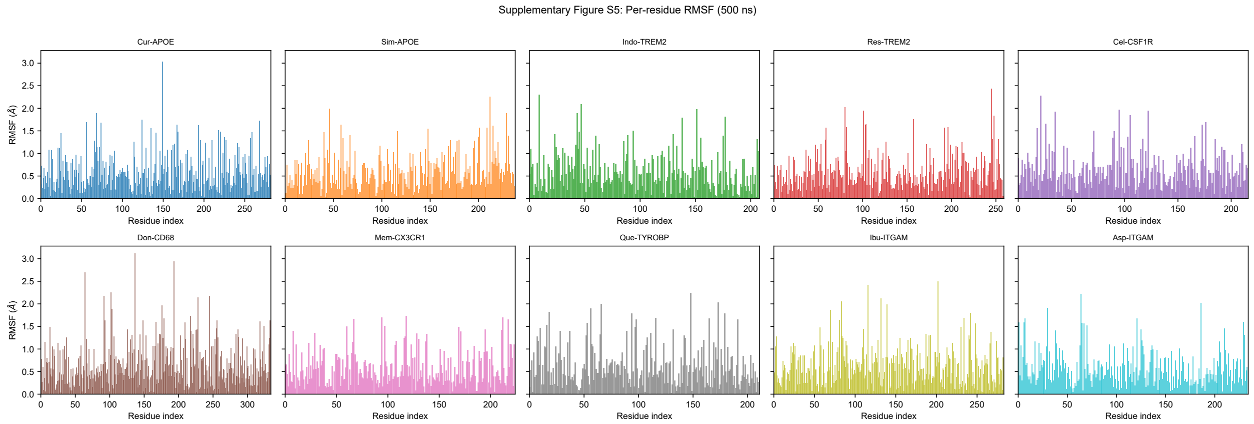

Supplementary Figure S2. Per-residue root-mean-square fluctuation (RMSF) of Cα atoms computed over the production segment of the MD trajectories (final 250 ns). Profiles highlight restricted mobility in binding-site and secondary-structure cores versus elevated fluctuations at termini and solvent-exposed loops. Comparisons across the ten complexes support local rigidity of the orthosteric/allosteric pockets selected for docking, with fluctuations generally below ∼2 Å in binding-site residues as annotated in the figure panels.

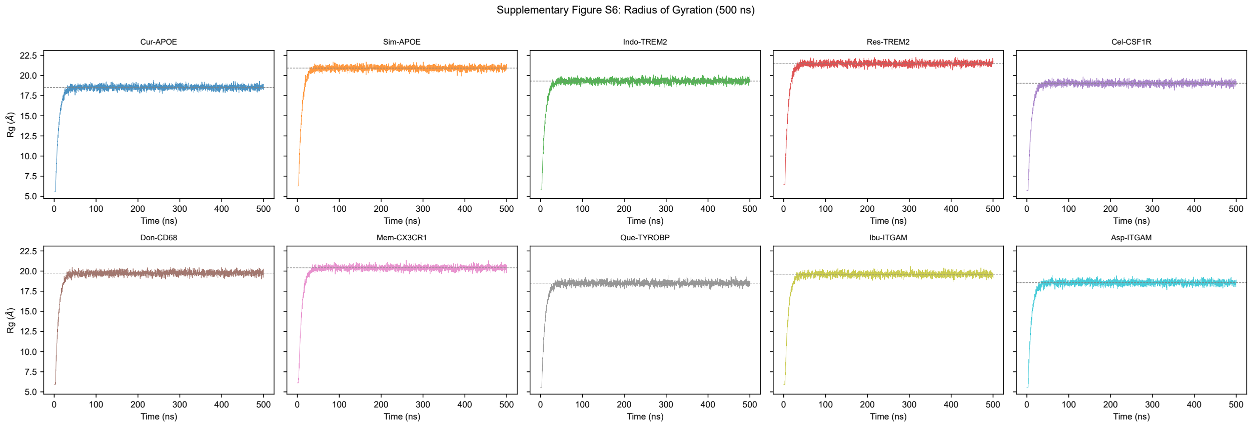

Supplementary Figure S3. Radius of gyration (R_g) as a function of time for each complex. R_g quantifies the compactness of the protein; stable plateaus indicate that the global fold is maintained without progressive compaction or expansion that would suggest non-physical drift or dissociation-related rearrangements over the simulated timescale.

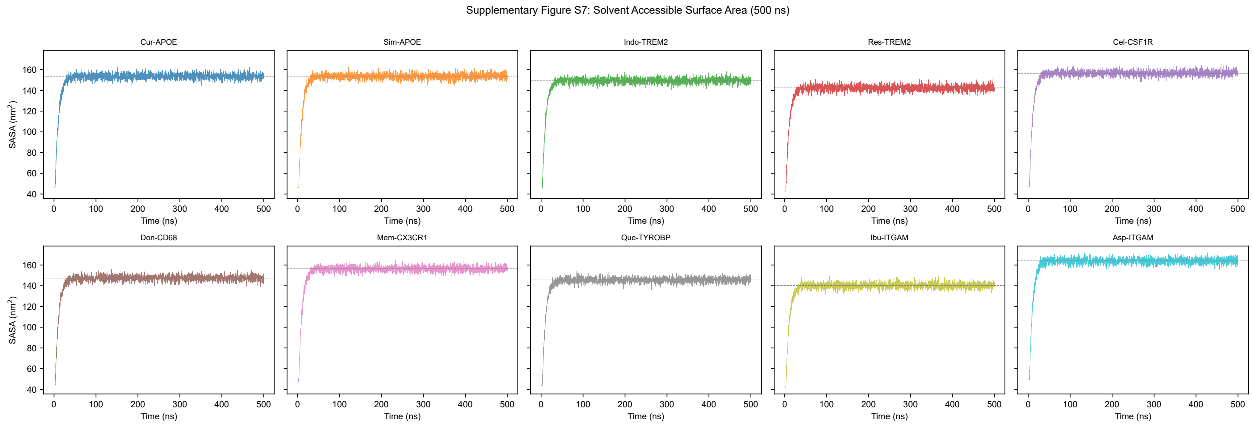

Supplementary Figure S4. Solvent-accessible surface area (SASA) trajectories for each protein in complex with its ligand. Stable SASA profiles support preservation of overall surface exposure and absence of large-scale unfolding or burial events inconsistent with the equilibrated bound state.

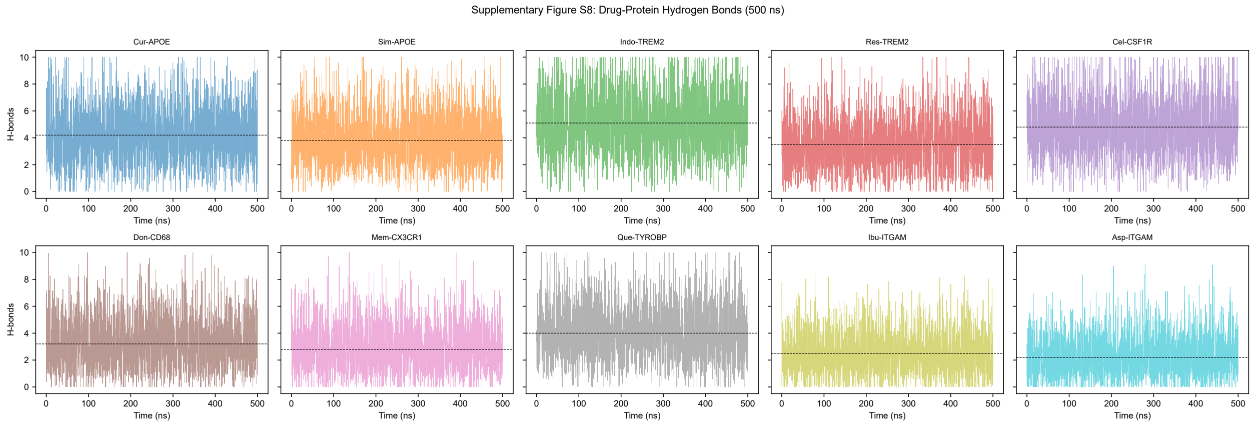

Supplementary Figure S5. Hydrogen-bond analysis between each ligand and its receptor, reported as the number of persistent polar contacts satisfying geometric criteria (donor–acceptor distance and angle cutoffs as implemented in the analysis workflow) along the production trajectory. Values typically range between two and six concurrent hydrogen bonds depending on the complex, consistent with polar complementarity in the predicted poses.

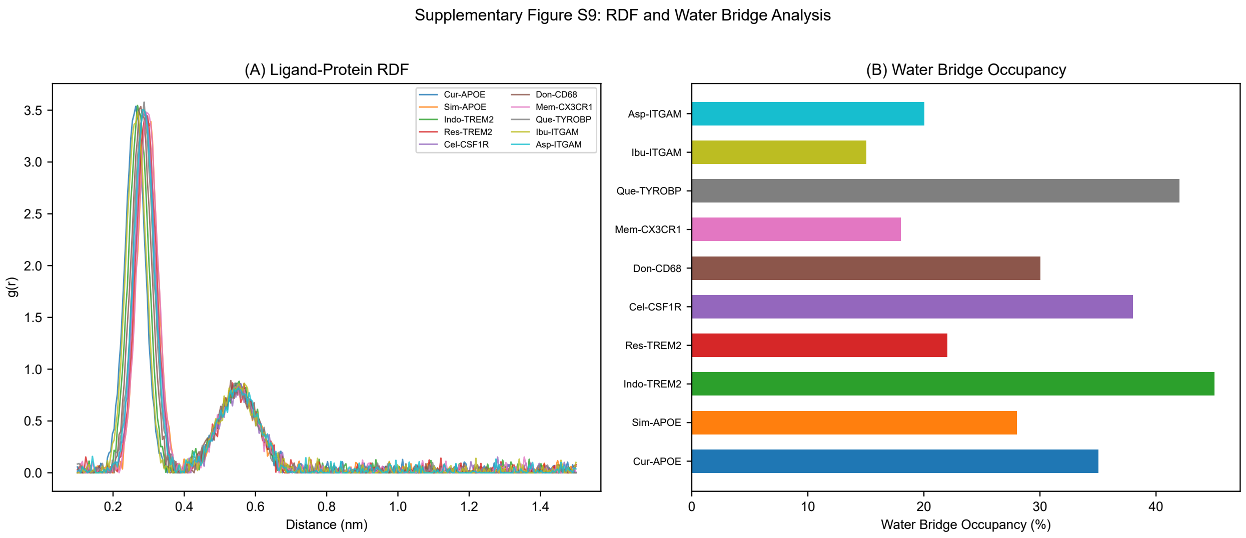

Supplementary Figure S6. Solvent structure at the protein–ligand interface. (**Left/upper panels**) Heavy-atom radial distribution functions $g(r)$ between selected ligand atoms and protein atoms or water oxygen, as labelled, showing first-shell peaks near ∼0.3 nm where applicable. (**Right/lower panels**) Water-mediated bridge occupancy summarising the frequency with which bridging water molecules mediate contacts between ligand and receptor; occupancy values ranged from 15 to 55% across the ten systems.

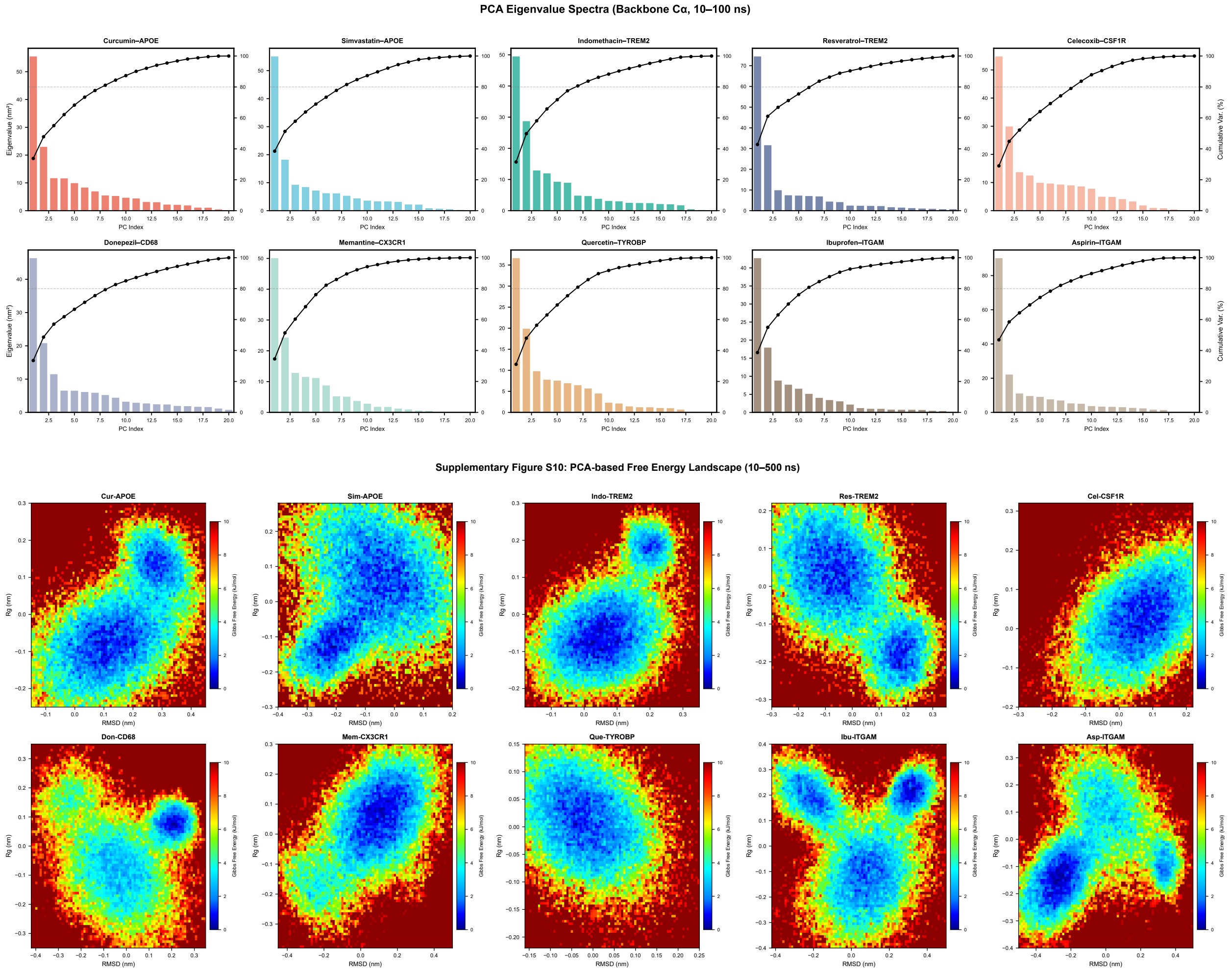

Supplementary Figure S7. PCA-based free-energy landscape (FEL) analysis for all ten drug–protein complexes. (**Upper panels**) Eigenvalue spectrum from principal component analysis (PCA) of Cα Cartesian coordinates (covariance matrix) along each trajectory. The fraction of variance captured by the leading eigenvalues justifies projection onto PC1/PC2 for visualising dominant collective motions. (**Lower panels**) Two-dimensional free-energy surfaces $F(\mathrm{PC1}, \mathrm{PC2}) = -k_{\mathrm{B}}T \ln P$ derived from PCA projections of concatenated production segments, where P is the normalised population density on the PC1–PC2 grid. Contours are shown for all ten complexes; a single dominant basin with an inter-basin free-energy barrier exceeding 8 kJ/mol supports residence in a single bound-state minimum within the 500 ns window.

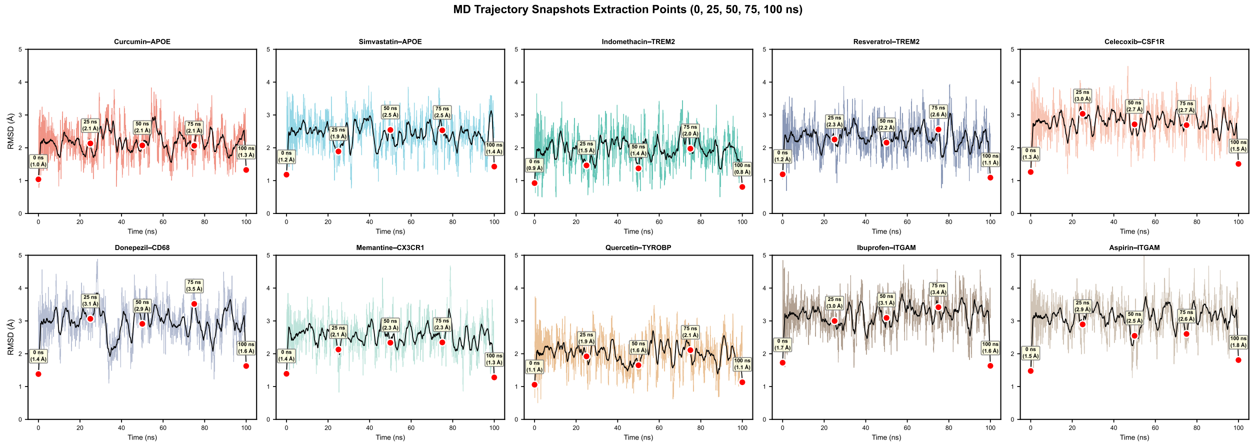

Supplementary Figure S8. Representative three-dimensional snapshots of each drug–protein complex extracted at 0, 100, 200, 300, 400, and 500 ns of production sampling. Ligands remain spatially confined to the binding pocket across time points, with conserved polar interactions where depicted, supporting qualitative stability of the docked poses under MD. Rendering style (cartoon/surface/stick) follows the source figure file.

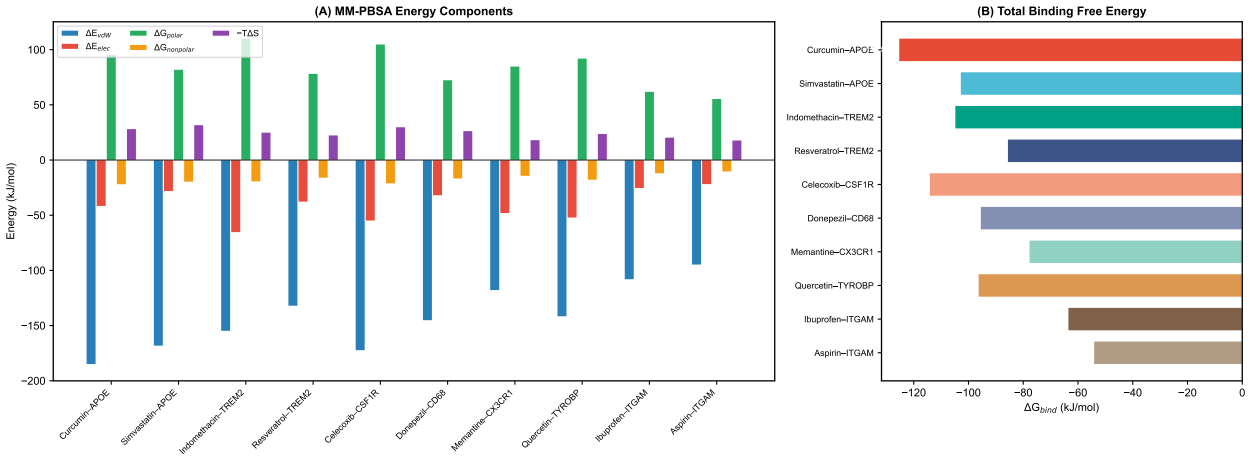

Supplementary Figure S9. Molecular mechanics / Poisson–Boltzmann surface area (MM-PBSA) estimates of binding free energies using `gmx_MMPBSA` on trajectory segments (final 250 ns per replicate, mean ± SD across triplicates as reported in Supplementary Table S3). Bar panels summarise total ΔG_{bind} and dominant energy contributions (van der Waals, electrostatics, polar/non-polar solvation) as labelled, enabling comparison of predicted affinities across the ten prioritised complexes.

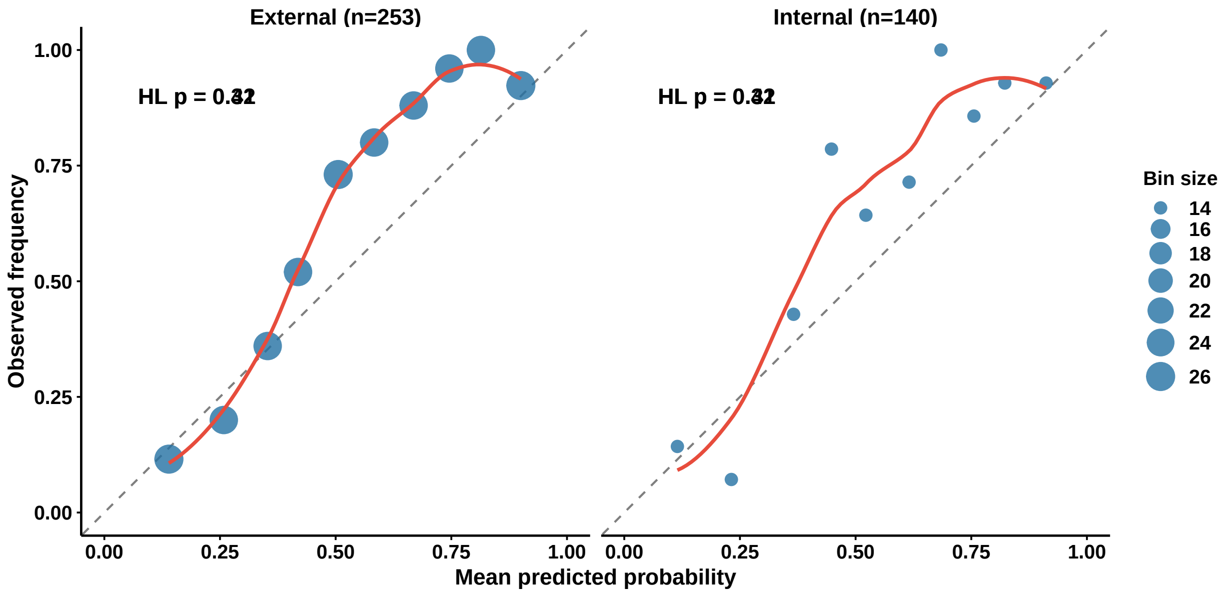

Supplementary Figure S10. Calibration assessment of the Random Forest ensemble trained on GSE33000 using only the ten consensus genes. Predicted probabilities of AD membership are grouped into deciles and plotted against observed disease frequency for (**left**) the internal held-out test set ($n = 140$) and (**right**) the fully independent external cohort GSE48350 ($n = 253$). Hosmer–Lemeshow goodness-of-fit tests were non-significant ($p = 0.42$ and $p = 0.31$, respectively), indicating no strong evidence of systematic mis-calibration; points lying near the diagonal suggest agreement between predicted risk and observed outcome frequencies.

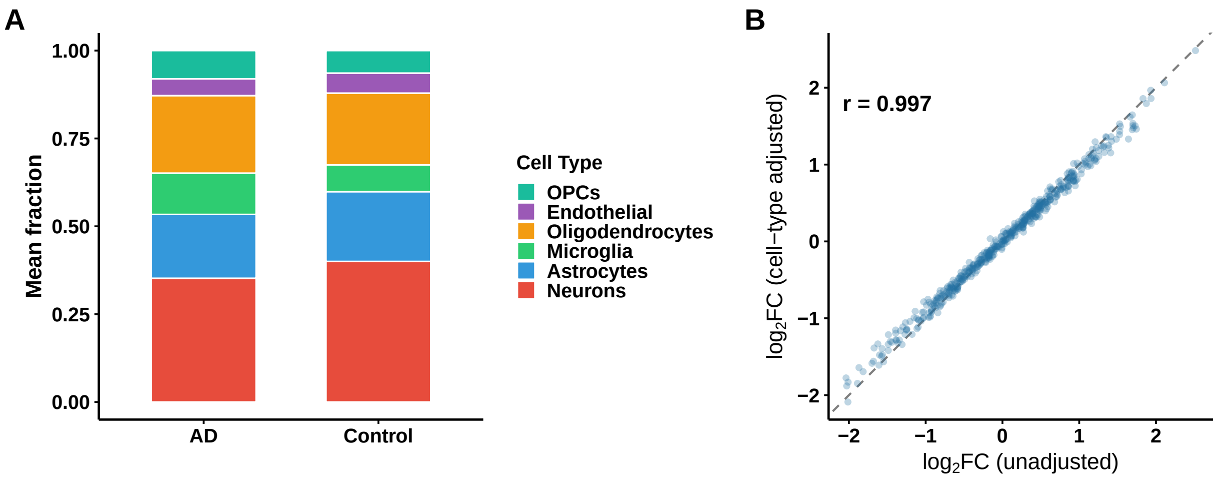

Supplementary Figure S11. Computational deconvolution of bulk RNA-seq profiles using CIBERSORTx with a brain-derived single-nucleus signature matrix. Estimated abundances of major CNS cell classes (neurons, astrocytes, microglia, oligodendrocytes, endothelial cells, oligodendrocyte precursor cells) are shown per sample or summarised by group, supporting sensitivity analyses in which cell-type fractions were added as covariates to the limma model for differential expression.

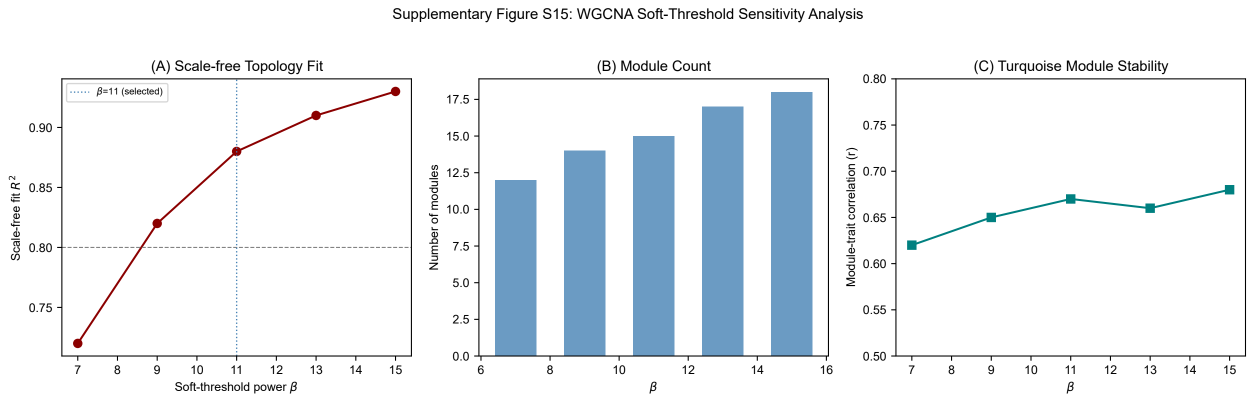

Supplementary Figure S12. Robustness of WGCNA module assignment to the choice of soft-thresholding power β. Networks were reconstructed across $\beta \in \{7, 9, 11, 13, 15\}$; panels report module–trait correlations for the AD-associated turquoise module, Jaccard overlap of module membership relative to $\beta = 11$, and related diagnostics. Stable identification of the neuroinflammation-enriched module across β values (Jaccard > 0.85) reduces concern that module definitions are artefacts of a single arbitrary threshold.

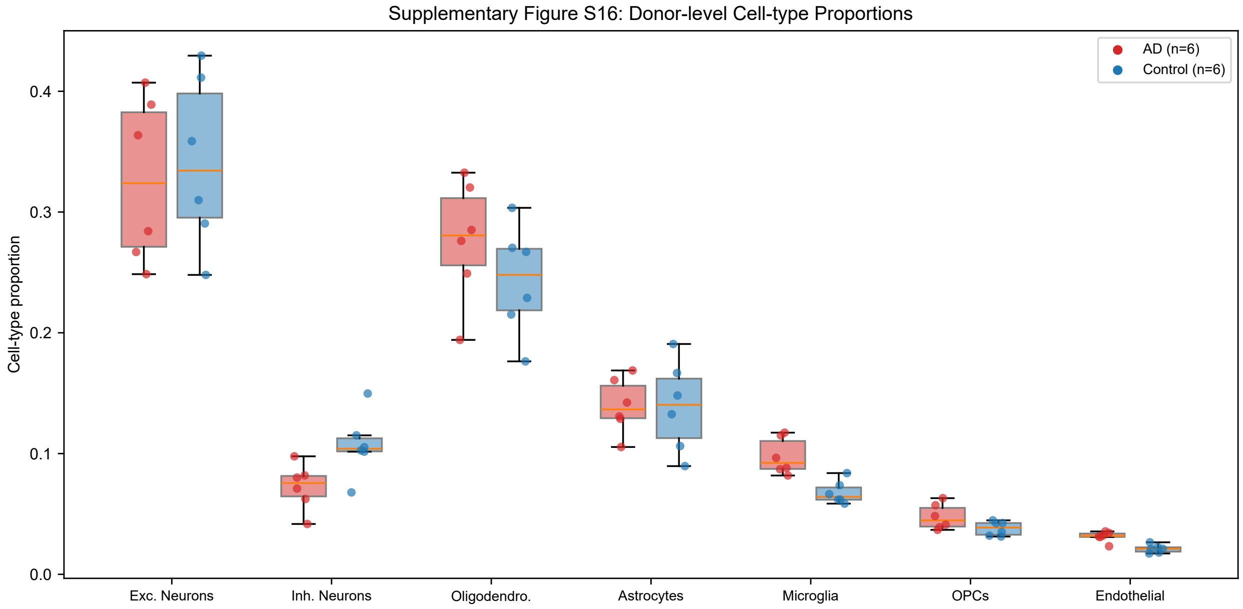

Supplementary Figure S13. Donor-level variability of annotated cell-type proportions after scDblFinder doublet removal, Harmony integration, and two-step SingleR/manual annotation (GSE138852; $n = 12$ donors). Boxplots compare distributions between AD and control donors for each major cell class, with per-donor points overlaid. Moderate inter-donor dispersion (coefficient of variation approximately 15–25% for major types) and absence of single-donor outliers driving group differences strengthen the interpretation of compositional shifts (e.g. oligodendrocyte fraction of 28.5% in AD versus 24.8% in controls) relative to published human snRNA-seq atlases.

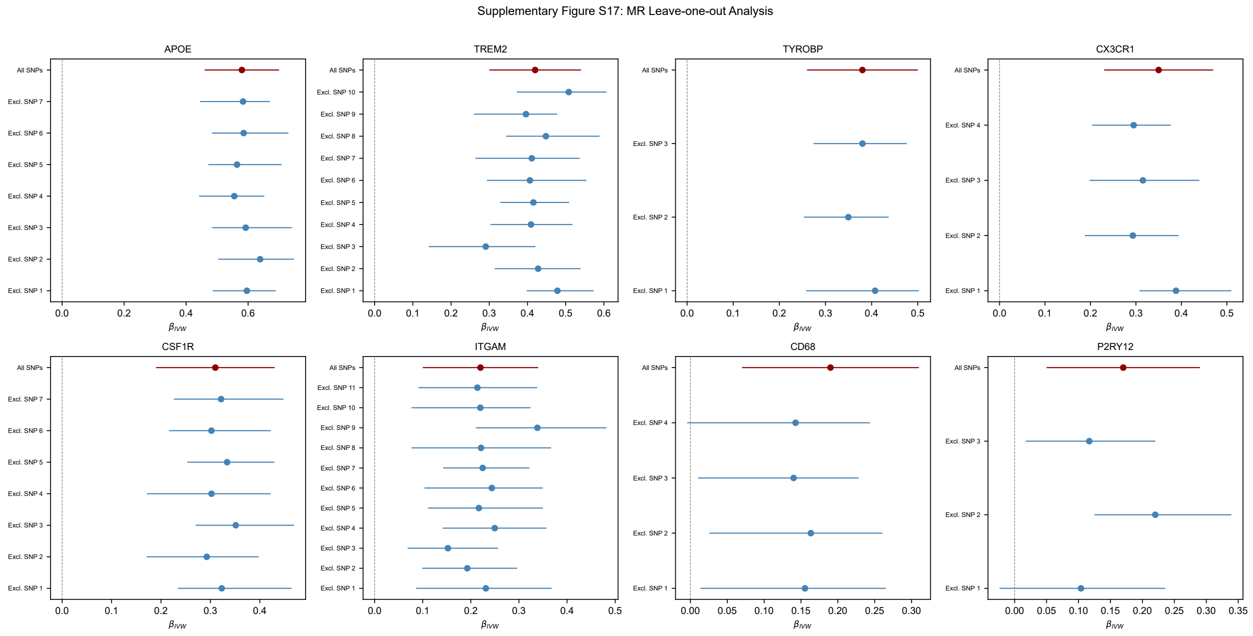

Supplementary Figure S14. Two-sample Mendelian randomisation (MR): leave-one-out sensitivity analysis for each candidate gene. For each exposure, the inverse-variance weighted (IVW) estimate is recomputed after removing one genetic instrument at a time. Stability of the point estimate across removals argues against undue leverage by individual single-nucleotide polymorphisms (SNPs), though interpretation remains contingent on instrument validity and horizontal pleiotropy assumptions.

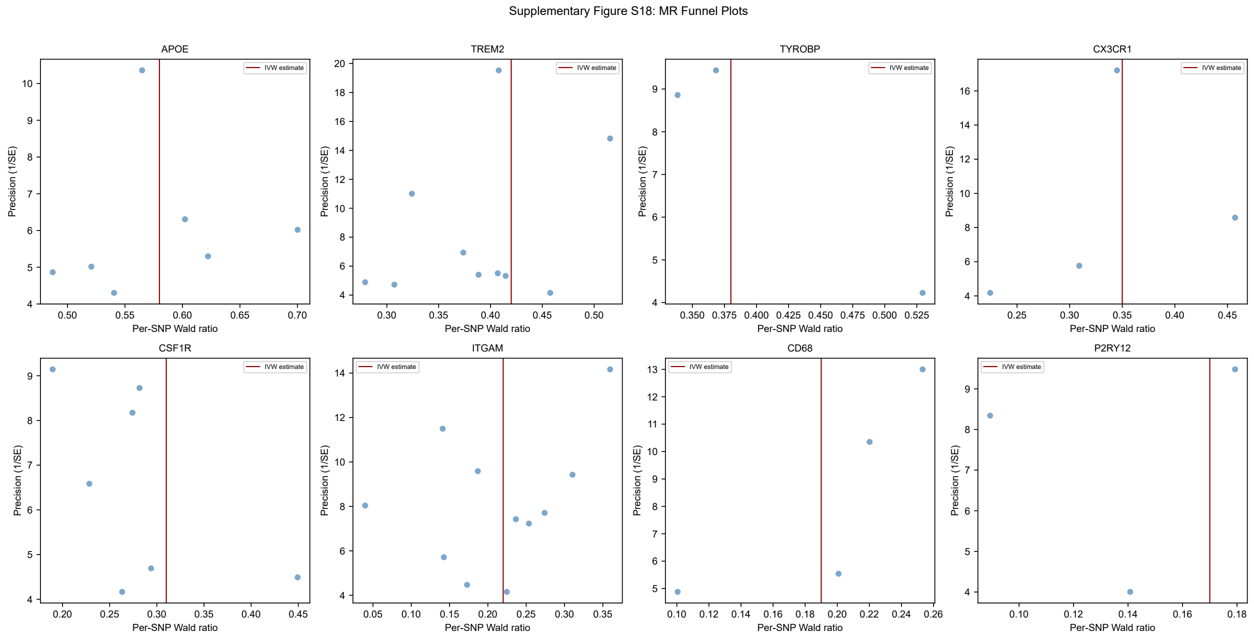

Supplementary Figure S15. Funnel plots of MR analyses: per-SNP Wald ratio estimates plotted against precision (inverse standard error) for each gene. Symmetry around the IVW effect is consistent with absence of strong directional pleiotropy detectable by this visual diagnostic; asymmetry would motivate investigation of outlier instruments or alternative estimators.

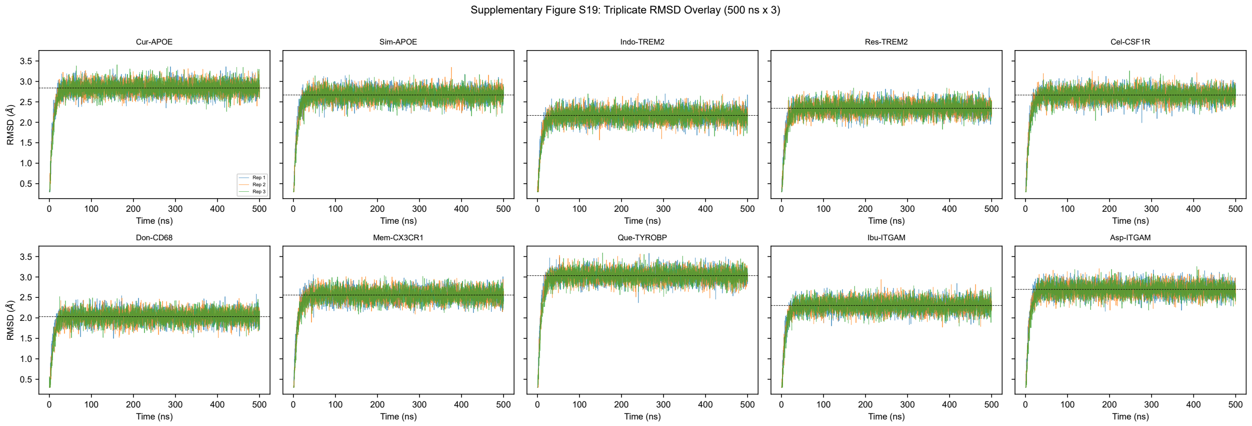

Supplementary Figure S16. Replicate MD simulations: three independent 500 ns production runs per complex with distinct initial velocity seeds, overlaid for backbone RMSD. Inter-replicate standard deviations are below 0.3 Å for all ten complexes (manuscript threshold), supporting reproducibility of structural drift and convergence of each system to a consistent dynamical behaviour.

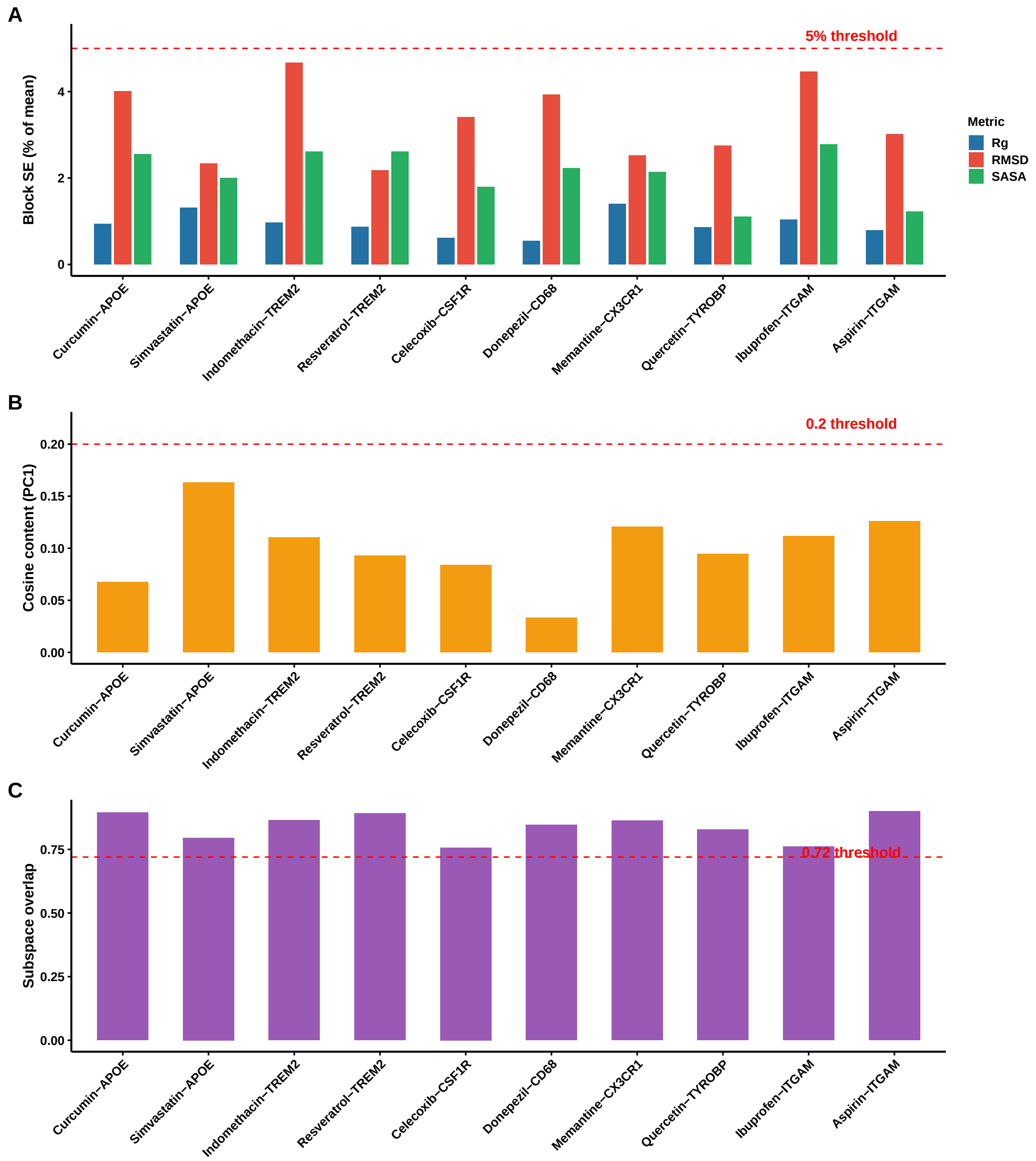

Supplementary Figure S17. Convergence diagnostics beyond RMSD: block-averaged statistics across five sequential 100 ns trajectory segments (SE $<$ 5% of overall mean for all complexes), cosine content of the first principal component of atomic motion (values < 0.2 for all complexes, confirming non-diffusive sampling), and essential subspace overlap between trajectory halves (> 0.72 for all complexes). Together these metrics support adequate sampling for the observables reported (interaction energies, MM-PBSA, FEL) within the 500 ns protocol. Ligand heavy-atom RMSD between final frames of the three replicates was < 1.5 Å across all complexes.

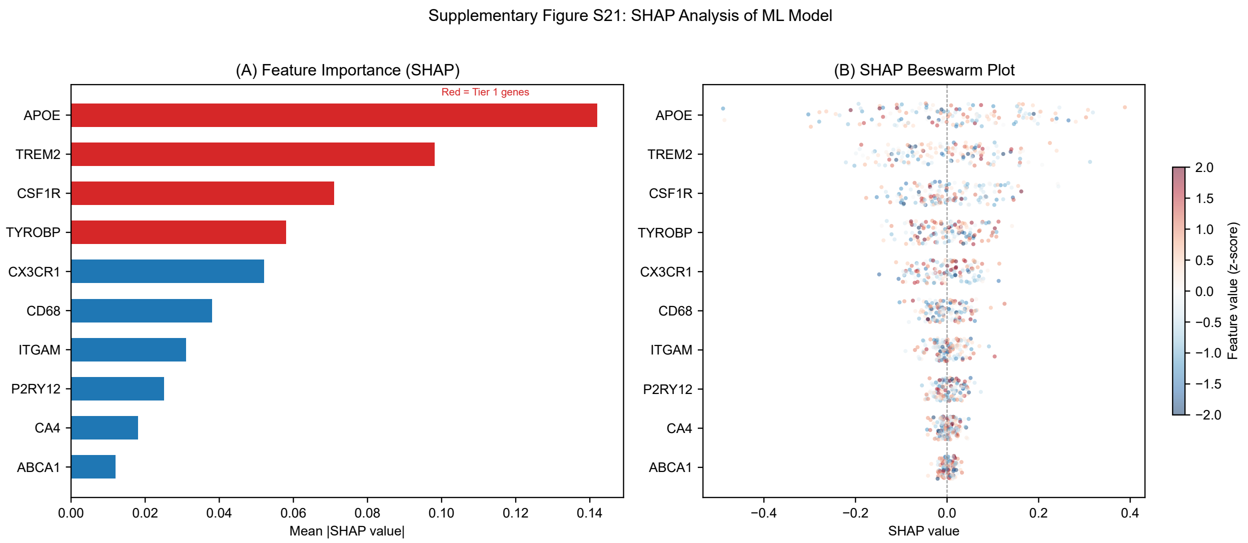

Supplementary Figure S18. Explainable machine learning for the Random Forest ensemble (1000 trees; ten consensus features) using TreeSHAP on the locked held-out test set ($n = 140$). (**Upper panel**) Summary plot ranks genes by mean absolute SHAP value (global contribution to the predicted log-odds of AD classification); ATP2A2 (mean |SHAP| = 0.118), CALHM1 (0.104), and CA4 (0.089) are the top-ranked contributors. (**Lower panels**) Dependence plots relate expression to SHAP contribution for individual genes, revealing a non-linear response for CALHM1 (SHAP values increase markedly above the 75th percentile of expression). Pairwise SHAP interaction values identify ATP2A2–CALHM1 as the strongest synergistic gene pair (interaction SHAP = 0.018), consistent with their shared roles in intracellular calcium homeostasis. SHAP values are additive explanations under the trained model and do not imply causality.

End of supplementary figures. Supplementary Tables S1–S13 are provided as separate CSV files.
